# Long-term simulated microgravity alters gut microbiota and metabolome in mice

**DOI:** 10.3389/fmicb.2023.1100747

**Published:** 2023-03-24

**Authors:** Lu Yuan, Rong Zhang, Xinlou Li, Caiyun Gao, Xiangnan Hu, Safdar Hussain, Linlin Zhang, Moye Wang, Xiaoyu Ma, Qiuxia Pan, Xiaotong Lou, Shaoyan Si

**Affiliations:** ^1^Department of Medical Research, PLA Strategic Support Force Medical Center, Beijing, China; ^2^Center for Applied Molecular Biology, University of the Punjab, Lahore, Pakistan; ^3^Department of Traditional Chinese Medicine, PLA Strategic Support Force Medical Center, Beijing, China

**Keywords:** associations, metabolic pathways, intestinal metabolites, intestinal microbiota, simulated microgravity, long-term

## Abstract

Spaceflight and microgravity has a significant impact on the immune, central nervous, bone, and muscle support and cardiovascular systems. However, limited studies are available on the adverse effects of long-term microgravity on the intestinal microbiota, metabolism, and its relationships. In this study, a ground-based simulated microgravity (SMG) mouse model was established to evaluate the impact of long-term microgravity on gut microbiota and metabolome. After 8 weeks of SMG, alterations of the intestinal microbiota and metabolites were detected using 16S rRNA sequencing and untargeted metabolomics. Compared to the control, no significant differences in α-diversity were observed at weeks 2, 4 and 8. Nevertheless, there were clear differences in community structures at different time points. The phylum *Verrucomicrobia* significantly declined from 2 to 8 weeks of SMG, yet the relative abundance of *Actinobacteria* and *Deferribacteres* expanded remarkably at weeks 8. SMG decreased the genus of *Allobaculum* and increased *Bacteroides* significantly throughout the period of 8 weeks. Besides, Genus *Akkermansia*, *Gracilibacter*, *Prevotella*, *Odoribacter*, *Rothia*, *Sporosarcina*, *Gracilibacter*, *Clostridium,* and *Mucispirillum* were identified as biomarkers for SMG group. *Desulfovibrio_c21_c20, Akkermansia_muciniphila*, and *Ruminococcus_gnavus* dropped at week 2, which tend to recover at week 4, except for *Akkermansia_muciniphila*. *Bacteroides_uniformis* and *Faecalibacterium_prausnitzii* declined significantly, while *Ruminococcus_flavefaciens* and *Mucispirillum_schaedleri* elevated at week 8. Furthermore, intestinal metabolome analysis showed that 129 were upregulated and 146 metabolites were downregulated in SMG. Long-term SMG most affected steroid hormone biosynthesis, tryptophan, cysteine, methionine, arginine, proline metabolism, and histidine metabolism. Correlated analysis suggested that the potential beneficial taxa *Allobaculum, Akkermansia*, and *Faecalibacterium* were negatively associated with tryptophan, histidine, arginine, and proline metabolism, but positively with steroid hormone biosynthesis. Yet *Bacteroides, Lachnospiraceae_Clostridium, Rothia, Bilophila,* and *Coprococcus* were positively correlated with arginine, proline, tryptophan, and histidine metabolism, while negatively associated with steroid hormone biosynthesis. These results suggest that Long-term SMG altered the community of intestinal microbiota, and then further disturbed intestinal metabolites and metabolic pathways, which have great potential to help understand and provide clues for revealing the mechanisms of long-term SMG involved diseases.

## Introduction

Long-duration spaceflight and lunar missions are essential projects for the astronauts in the present and future (Williams et al., 2009). Microgravity, radiation, isolation, and immobilization are some of the factors that profoundly influence astronauts’ physiology during spaceflight (LaPelusa et al., 2021). Multiple shreds of evidence demonstrate that spaceflight has an impact the human immune system, central nervous system, bone and muscle support system, cardiovascular system, and microbiome (Crucian et al., 2016; Van Ombergen et al., 2017). The spaceflight-associated neuro-ocular syndrome has been documented in astronauts during long-duration spaceflights (Lee et al., 2020). During space flight, increased virulence and immune dysregulation may contribute to elevate risk of infectious diseases (Wilson et al., 2007)_._ Therefore, it becomes increasingly important to investigate the impact of specific environmental factors on long-term health in spaceflight.

Gut microbiota play a pivotal role in health and disease. Dysbiosis of gut microbiota is associated with several disorders, including inflammatory bowel disease, obesity, metabolomics, cardio cancer, and even neurodegenerative disorders (Weingarden and Vaughn, 2017; Lim, 2022; Paik et al., 2022; Park et al., 2022; SantaCruz-Calvo et al., 2022; Sorboni et al., 2022). Recent studies demonstrate that the human gut microbiota is sensitive to spaceflight. Previous analysis of the microbiome composition of crewmembers and participants in analog missions suggested that the composition and function of the gut flora were substantially affected by spaceflight and analog missions, including changes to alpha and beta diversity and microbial and host gene expression (Liu et al., 2020). In a long ground-based spaceflight project, MARS500, the human gut microbiota of six astronauts was inherently dynamic, capable of shifting between different steady states and some key microbial clusters showed conserved temporal dynamics (Turroni et al., 2017).

Microgravity is one of the major environmental risk factors for astronauts in spaceflight. In a 45-day head-down bed rest experiment, the urinary excretion of metabolites related to the deconditioning of bone, muscle, and gut flora changed dramatically (Chen et al., 2016). The model of hindlimb unloading in mice or rats, a well-established ground-based simulated microgravity model, was commonly used to mimic specific physiological effects (Chelyshev et al., 2014; Kulikova et al., 2017; Marzuca-Nassr et al., 2019). A recent study illustrated that simulated microgravity suppresses the p38 mitogen-activated protein kinase (MAPK) pathway-mediated innate immune response to bacterial infection and induces gut microbiota dysbiosis. While probiotics VSL#3 alleviated gut microbiota dysbiosis and led to the inhibition of innate immunity (Wang et al., 2020). These human and animal studies indicate the impact of short-term simulated microgravity on the intestinal flora.

Gut microbiota derived-metabolites play critical roles in maintaining health (Wang and Zhao, 2018). After 21 days of simulated weightlessness, the intestinal metabolic profile in rats is significantly altered, mainly participating in pyrimidine metabolism and pentose and glucuronate interconversions metabolism (Jin et al., 2019). This project also indicated that the differential metabolites were associated with the changes of the intestinal microbiota and disruption of immunological characteristics (Jin et al., 2019). The interaction between host physiology and microbial metabolites is complex and the nonconformity is attributed to microbial, environmental and host sources (Krautkramer et al., 2021). Therefore, short-term microgravity probably disturbs the intestinal flora, further leading to alteration of the metabolites. As the remarkably increased space missions and the essential role of gut homeostasis in astronaut physiology, it is necessary to reveal the influence of long-term weightlessness on microbiota and metabolites. However, studies focused on the adverse effects of long-term microgravity on intestinal microbiota, metabolome, and their associations are limited. Here, we established a hindlimb unloading mouse model of microgravity and performed multi-omics analysis of 16S rRNA sequencing and untargeted metabonomic technology to access the impact of 8 weeks of SMG on the intestinal microbiota, metabolites, and participating pathways. Then, we further analyze and their associations in detail. Our work will provide a better understanding of the impact of long-term microgravity on intestinal homeostasis, which provides clues for further revealing the mechanisms of long-term SMG involved diseases.

## Materials and methods

### Animals and SMG model construction

Sixteen C57BL/6 mice (female, 6–8 weeks) of SPF grade were purchased from SiPeiFu Biotechnology Co. Ltd., Beijing, China. All mice were kept in separate cages in a temperature-controlled environment. The light–dark cycle was 12/12 h, and they had free access to food and water. After 1 week of adaptive feeding, mice were randomly divided into two groups, the control group (Control) and the simulated microgravity group (SMG), each group contained eight mice. Hindlimb suspension of mice by the tail model was followed to create a ground-based model of microgravity (Globus and Morey-Holton, 2016). The mice were suspended by tail with hindlimbs suspended at 15–30° horizontally, ensuring that the hindlimbs do not touch the cage floor. Mice in the control group were raised in identical cages without hindlimb unloading. Thirty-six fecal samples from 6 mice of each group were collected at the end of week 2, 4, and 8, and stored at −80°C until used for 16S rRNA gene sequencing. Mice were sacrificed at the end of week 8. Then, cecal contents from each mouse were obtained and saved at −80°C until used for metabolic analysis. All efforts were made to minimize the discomfort of the animals. The whole experimental design is shown in revised Supplementary Figure S1.

### DNA extraction and 16S rRNA gene sequencing

Total bacteria DNA was extracted from approximately 220 mg of stool samples using the CTAB method following the standard protocol and then sequencing libraries were created. Briefly, the V3-V4 region of the 16S rRNA genes was amplified using specific primer (341F, 5ˊ-CCTAYGGGRBGCASCAG-3ˊ, 806R, 5ˊ-GGACTACNNGGGTAT CTAAT-3ˊ) with the barcode. The sequencing libraries were generated using TruSeq® DNA PCR-Free Sample Preparation Kit (Illumina, USA) according to the manufacturer’s instructions, and index codes were added. The library quality was assessed on the Qubit@ 2.0 Fluorometer (Thermo Scientific). Finally, the library was sequenced on an Illumina NovaSeq platform and 250 bp paired-end reads were generated.

### Sequence analysis

The analysis was conducted by following the “Atacama soil microbiome tutorial” of Qiime2 along with customized program scripts.^1^ Briefly, raw data FASTQ files were imported into the format which could be operated by the QIIME2 system. Demultiplexed sequences were quality filtered, trimmed, de-noised, and merged. Then the chimeric sequences were identified and removed using the QIIME2 dada2 plugin to obtain the amplicon sequence variant feature table (ASV) (Callahan et al., 2016). To generate the taxonomy table, the QIIME2 feature-classifier plugin was then used to align ASV sequences to a pre-trained GREENGENES 13_8 99% database (Bokulich et al., 2018). Kruskal–Wallis and LEfSe were employed to identify the bacteria with different abundance among samples and groups. Alpha diversity indices were calculated using qiime2 diversity, with the observed OTU to calculate species richness and Shannon index to calculate species diversity. Beta diversity distance measurements were performed to investigate the structural variation of microbial communities across samples and then visualized *via* nonmetric multidimensional scaling (NMDS). Co-occurrence analysis was performed by calculating Spearman’s rank correlations between predominant taxa and the network plot was used to display the associations among taxa.

### Metabolomics profiling

Fecal samples (100 mg) were individually grinded with liquid nitrogen and the homogenates were resuspended with prechilled 80% methanol by vigorous vortexing. The samples were incubated on ice for 5 min and then centrifuged at 15,000 g for 20 min at 4°C. Some of the supernatant was diluted to a final concentration of 53% methanol by LC–MS grade water. The samples were transferred to a new Eppendorf tubes and centrifuged at 15,000 g, 4°C for 20 min. Finally, the supernatant was injected into the LC–MS/MS system for analysis (Want et al., 2013).

UHPLC–MS/MS analysis was performed using a Vanquish UHPLC system (Thermo Fisher, Germany) coupled with an Orbitrap Q Exactive^TM^ HF mass spectrometer (Thermo Fisher, Germany) in Novogene Co., Ltd. (Beijing, China). Samples were injected onto a Hypesil Gold column (100 × 2.1 mm, 1.9 μm) using a 17-min linear gradient at a flow rate of 0.2 ml/min. The eluents for the positive polarity mode were eluent A (0.1% FA in Water) and eluent B (Methanol).The eluents for the negative polarity mode were eluent A (5 mM ammonium acetate, pH 9.0) and eluent B (Methanol). Q Exactive^TM^ HF mass spectrometer was operated in positive/negative polarity mode with spray voltage of 3.5 kV.

### Metabolomic data processing and analysis

The raw data files generated by UHPLC–MS/MS were processed using the Compound Discoverer 3.1 (CD3.1, Thermo Fisher) to perform peak alignment, peak picking, and quantitation for each metabolite. The normalized data were used to predict the molecular formula based on additive ions, molecular ion peaks, and fragment ions. And then peaks were matched with the mzCloud,^2^ mzVaultand MassListdatabase to obtain the accurate qualitative and relative quantitative results. Statistical analyses were performed using the statistical software (Rohart et al., 2017). When data were not normally distributed, normal transformations were attempted by using area normalization method. Random Forest machine learning analysis was constructed through tools of bioincloud.tech and Mean Decrease Accuracy is used to measure the importance of metabolites in distinguishing groups in random forests. These metabolites were annotated using the KEGG database, HMDB database, and LIPIDMaps database. Furthermore, MetOrigin database^3^ was used to discriminate the origins of identified metabolites.

### Statistical analysis

Statistical analysis of the results was performed by using the statistical software R (R version R-3.4.3) and GraphPad Prism 7.0 software. Statistical significance between SMG and control groups was evaluated by Mann–Whitney test and Kruskal–Wallis test. Differences between the groups were considered statistically significant at the 5% level (*p* < 0.05). All *p* -values for the statistical tests of metabolite variations in the two groups were corrected for multiple testing using Benjamini-Hochberg false discovery rates (FDR) method. Significant differences were indicated by *p < 0.05, **p < 0.01, ***p < 0.001. Correlations between the relative abundance of differential bacteria (*p* < 0.05, average relative adundance˃0.01%) and the concentration of major metabolites (FDR < 0.05) involving in major differential metabolic pathways were computed with Spearman’s test using Oebiotech tools^4^ and MetaboAnalyst 5.0.^5^

## Results

### Dynamic changes in community composition under the long-term simulated microgravity

**Figure 1:**
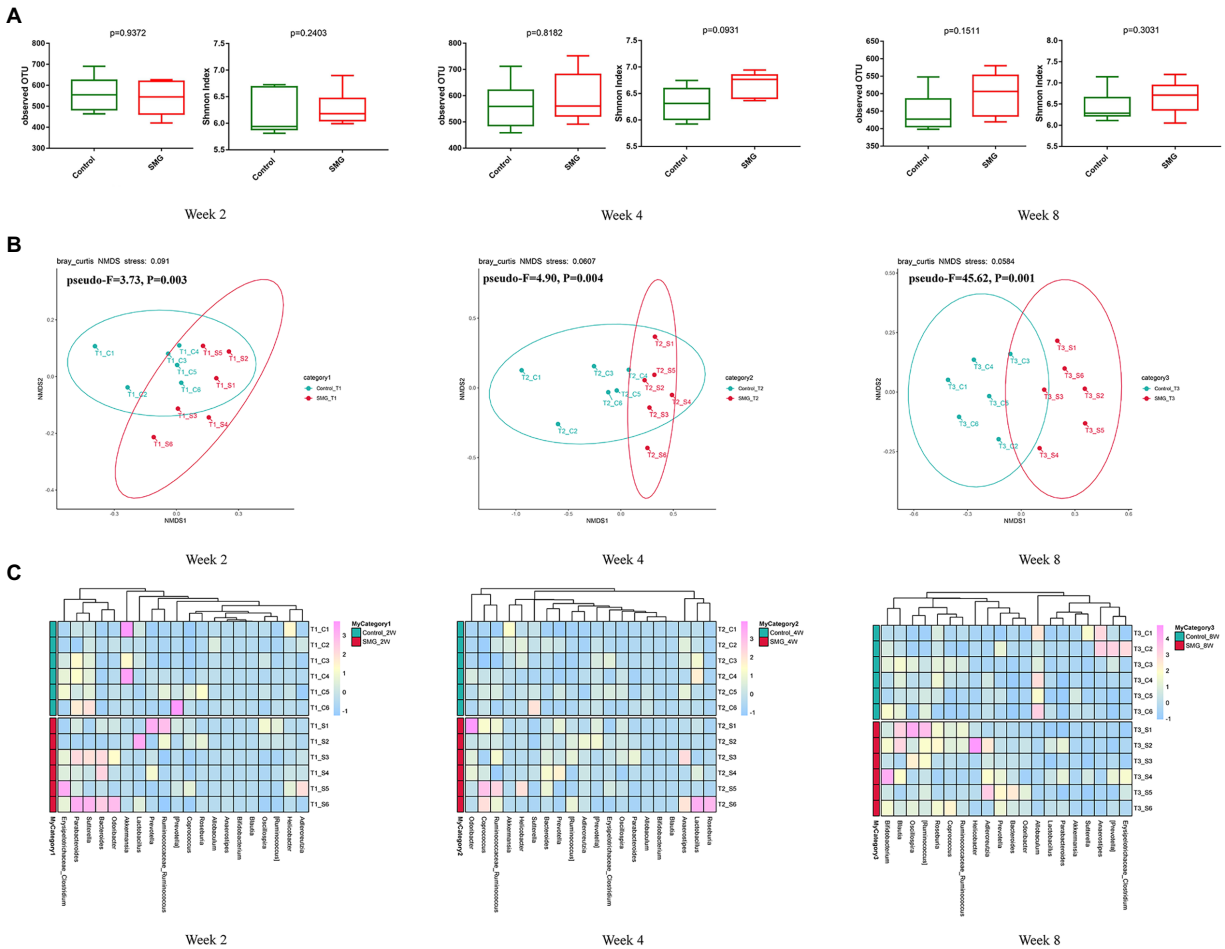
Bacterial diversity analysis of the gut microbiota in two groups during SMG treatment. **(A)** Alpha diversity metrics (observed OTU and Shannon indices) with respect to SMG and control at week 2, 4 and 8. **(B)** Beta diversity of the gut microbiota in two groups at week 2, 4 and 8. **(A)** The NMDS plot of gut microbiota from the control and SMG group at three time point intervals. **(C)** Heatmap of the relative abundance of top 20 bacterial genera at three time points.

We performed the V3-V4 region of the 16S rRNA gene sequencing to assess whether the long-term SMG altered the intestinal flora at different time points after 8 weeks of SMG. The 36 fecal samples yielded a total of 3,070,422 raw reads corresponding to 7,719 OTUs. The data were normalized by established a depth of 63,210 reads per sample., Alpha diversity analysis showed there were no significant differences in the indices of observed OTU and Shannon index (*p* > 0.05, Figure 1A) compared to the control mice at weeks 2, 4 and 8. These results suggested that SMG did not affect the richness and diversity of gut microbiota during the 8 weeks of SMG. Beta diversity reveals that SMG had a statistically significant effect on community composition and structure at weeks 2, 4, and 8 (*p* < 0.01, Figures 1B,C). Non-metric multidimensional scaling (NMDS) based on the Bray–Curtis distance revealed a significant differences in microbial structure between the control group and the SMG group (*p* < 0.01, Figure 1B). Better separation of SMG and control mice was observed at the genus level throughout the 8 weeks of SMG (Figure 1C). In addition, the co-network analysis showed that the microbial associations differed between the SMG and the control groups at different time points (Supplementary Figure S3). For example, the relative abundance of *Akkermansia* is positively related to the beneficial bacteria *Lactobacillus* and negatively associated with *Bacillus* in the control mice at week 2. However, the relative abundance of *Akkermansia* is negatively relative with *Helicobacter* in SMG mice. Moreover, the difference between the two groups seemed more apparent in week 8.

### The most affected bacterial populations at different time points

**Figure 2:**
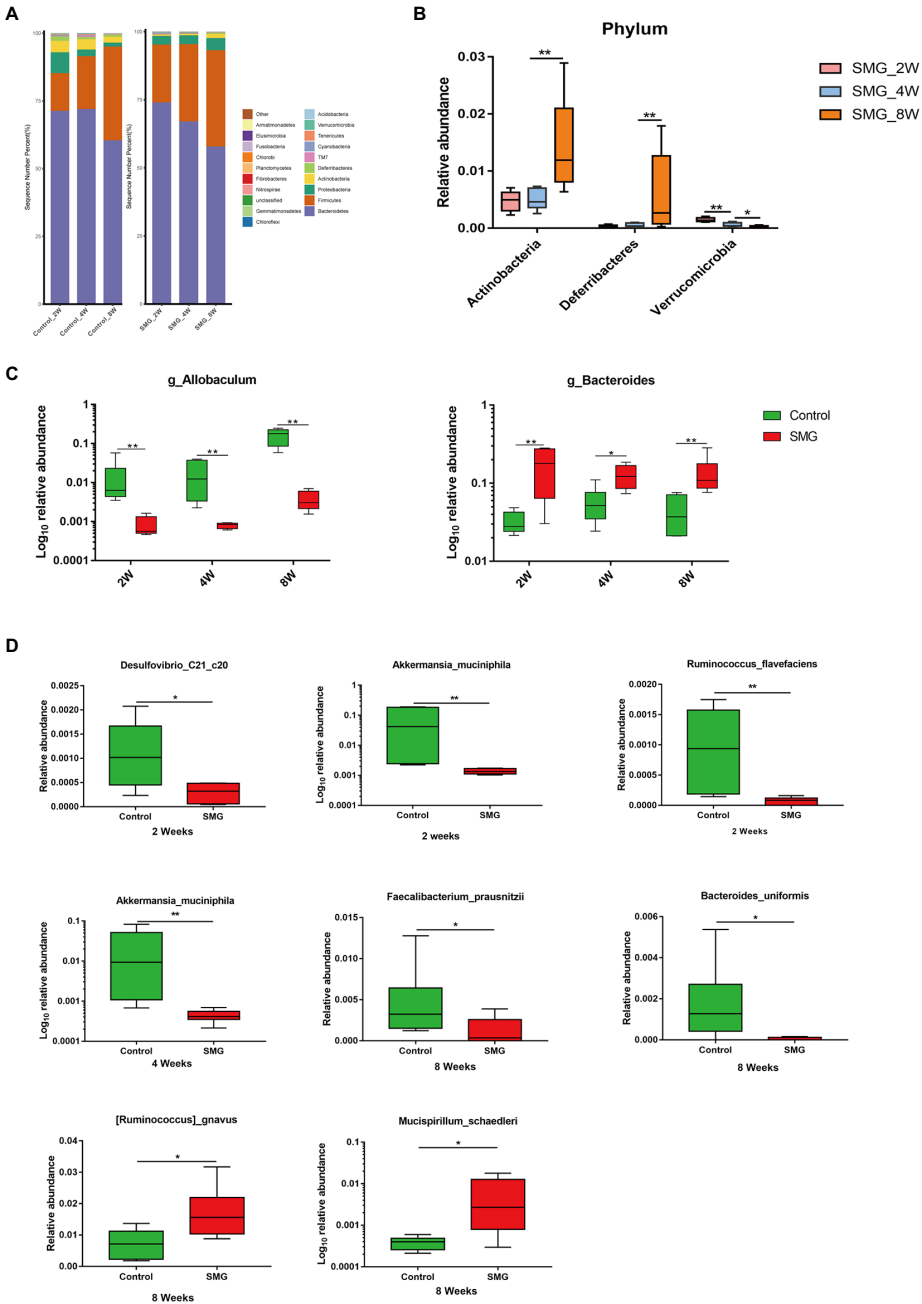
Taxonomic characterization of the gut microbiota in SMG mice at week 2, 4 and 8. **(A)** The column chart of the relative abundances of species in two groups the at phylum level. Differential bacteria in feces at phylum **(B)**, genus **(C)**, and species level **(D)**. *p < 0.05, **p < 0.01.

**Figure 3:**
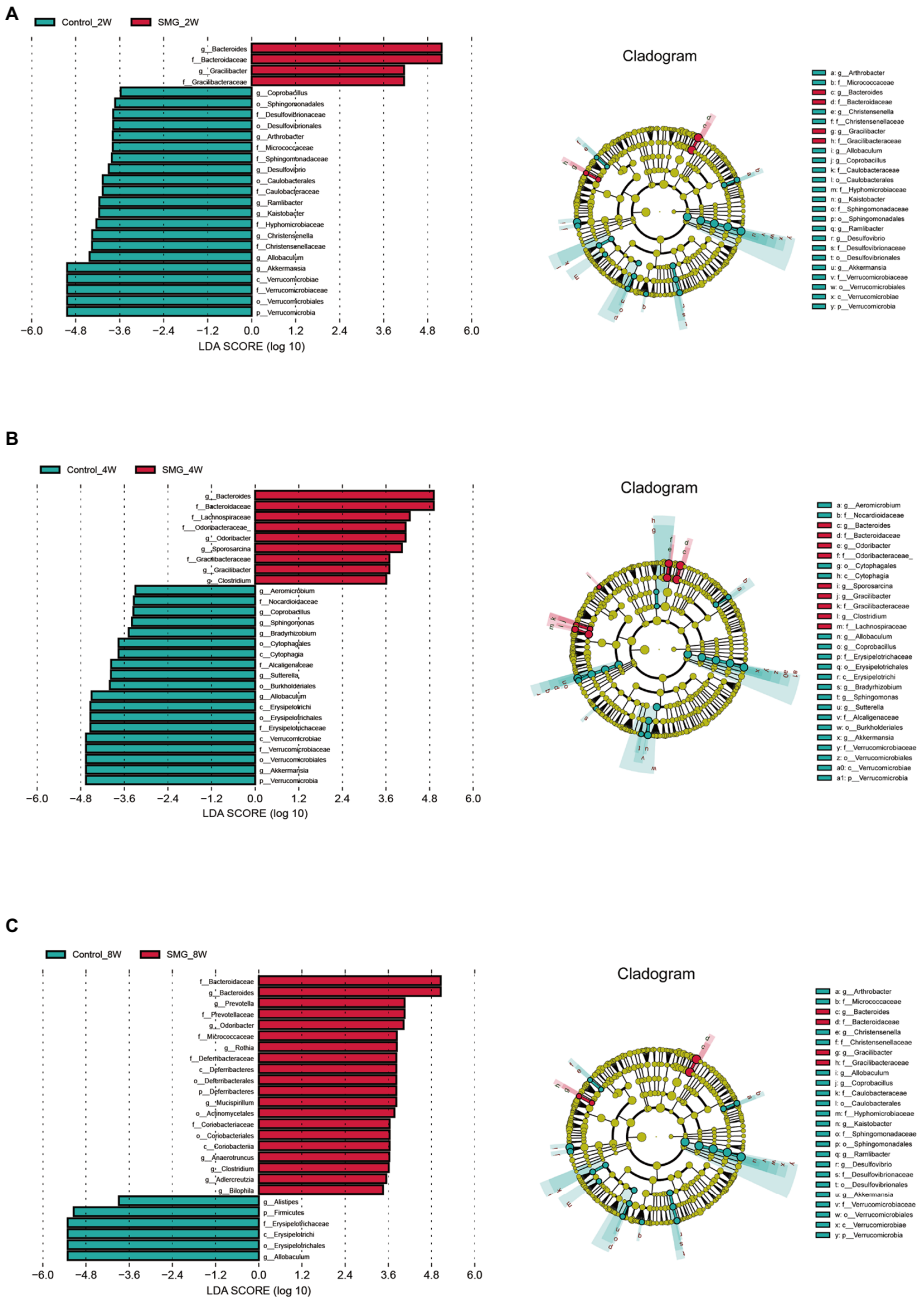
Microbial biomarkers associated with the SMG at week 2 **(A)**, 4 **(B)**, 8 **(C)**. Linear discriminant analysis (LDA) effect size (LEfSe) methods were used to identify the most deferentially abundant taxa between two groups at three time point. The cladogram of bacteria with larger effect size in the SMG group is shown in red, and bacteria with larger effect size in the control group are shown in green.

We also performed multivariate analysis to identify differential abundant taxa in SMG mice as compared to the control group at three-time points. At phylum level, fecal microbiota in both groups dominated by bacterial phyla *Firmicutes, Bacteroidetes,* and *Proteobacteria* (Figure 2A). The proportions of *Bacteroidetes* and *Firmicutes* significantly declined in the control group at week 8 (Supplementary Figure S2B). However, there were no significantly differences in abundance of *Bacteroidetes* and *Firmicutes* in the SMG groups (Supplementary Figure S2C). These results imply that long-term SMG may lead to spices belonging to Bacteroidetes prone to growth. SMG significantly decreased the relative abundance of *Verrucomicrobia* at a different time points, and *Actinobacteria* and *Deferribacteres* increased significantly at week 8 (Figure 2B). At the genus level, we observed that SMG showed a significant decrease in the relative abundance of *Allobaculum* and increased *Bacteroides* throughout the 8 weeks of SMG (Figures 2B,C, 3). At the species level, *Desulfovibrio_c21_c20*, *Akkermansia_muciniphila*, and *Ruminococcus_flavefaciens* were decreased considerably in SMG mice at week 2. At week 4, only *Akkermansia_muciniphila* were significantly decreased. At week 8, *Bacteroides_uniformis* and *Faecalibacterium_prausnitzii* were declined, while *Ruminococcus_gnavus* and *Mucispirillum_schaedleri* were significantly increased (Figures 2D). By performing LEFSe at the genus level, we identified two taxa (*Bacteroides* and *Gracilibacter*) characteristic of the SMG mice at week 2 and 8, while taxa such as *Akkermansia* and *Allobaculum* were enriched in the control group (Figure 3A). At week 4, we identified five taxa (Bacteroides, Odoribacter, Sporosarcina, Gracilibacter, and Clostridium) enriched in the SMG group (Figure 3B). Nine taxa were identified in SMG group at week 8, including Bacteroides, Prevotella, *Odoribacter*, *Rothia*, *Mucispirillum*, *Anaerotruncus*, *Clostridium*, *Adlercreutzia,* and *Bilophila* (Figure 3C). Together, these results suggested that *Bacteroides, Allobaculum*, and *Akkermansia* were the most affected genera throughout the simulated microgravity process. In line with previous studies (Wang et al., 2020; Sun et al., 2021), SMG could significantly reduce the relative abundance of beneficial bacteria, such as *Akkermansia* and *Allobaculum.* Our findings confirm that SMG induces a marked disturbance in the intestinal flora at different times, and longer SMG has a greater effect.

### Long-term SMG changed the intestinal metabolism and metabolic pathways

**Figure 4:**
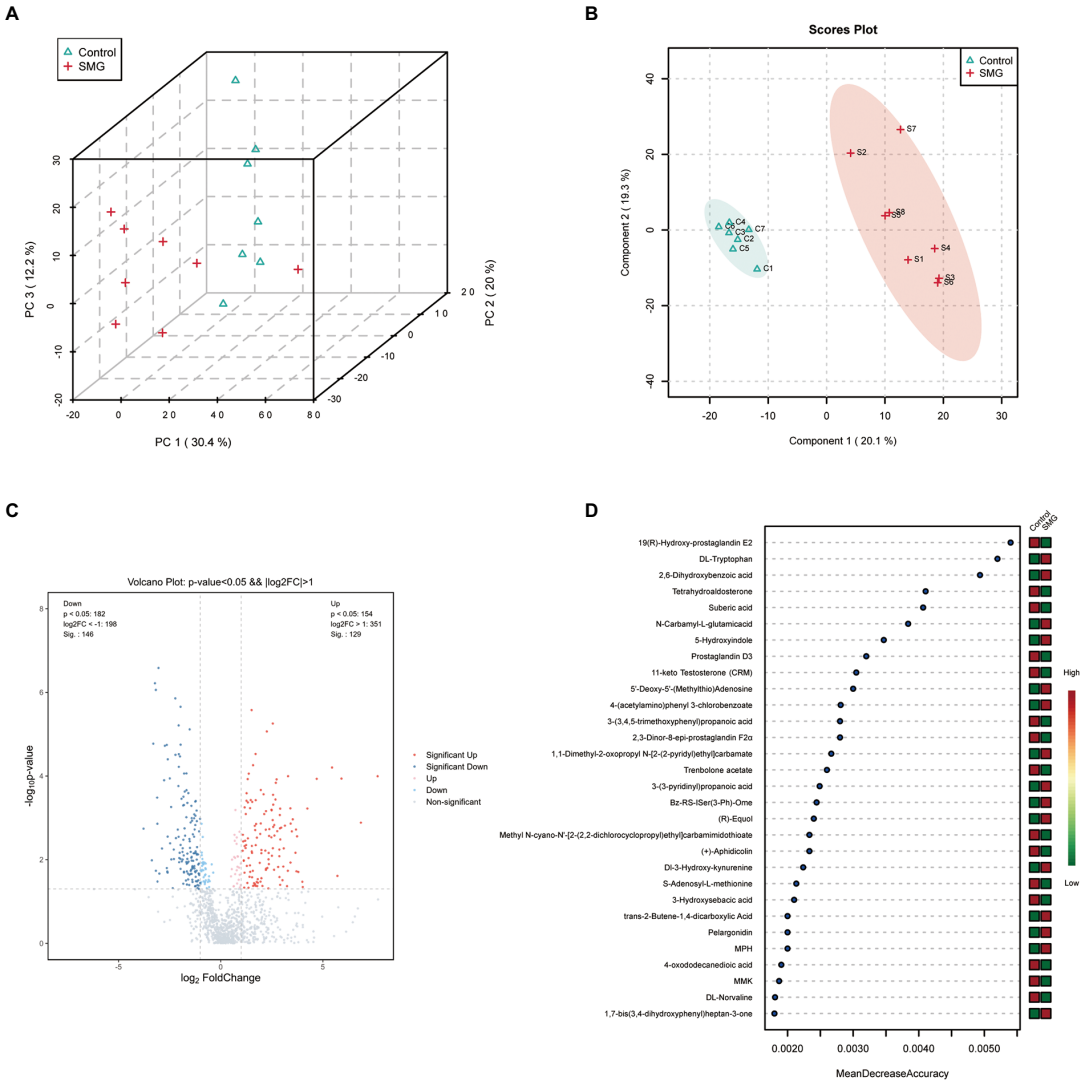
Long-term SMG alters intestinal metabolome profile. **(A)** PCA scores plots of intestinal metabolites based on the Bray–Curtis distance between SMG and control groups. **(B)** PLS-DA scores plots of intestinal metabolites between two groups. **(C)** Volcano plot of the differential metabolites between SMG and control groups. Each dot represents a specific metabolite. The red point means up-regulation, blue point means downregulation. **(D)** The top 30 named metabolites identified in random forests. The higher value of mean decrease accuracy, the higher the importance of the variable.

SMG treatment significantly altered the intestinal microbiota, raising our interest in investigating whether and how it could affect microbial metabolites, which could modulate circulating metabolites. We evaluated the intestinal metabolomics profiles from 15 samples of intestinal contents after 8 weeks of SMG. PCA and PLS-DA scores plots revealed clear significant differences in the composition of intestinal microbiota between the SMG and control groups (Figures 4A,B). A total of 1,193 metabolic features were identified across 15 samples. The volcano plots showed 275 features (23.05%) significantly changed in SMG (*p*<0.05) mice. Among them, 129 metabolites were upregulated and 146 downregulated (Figure 4C). On the super class level, the relative abundance of organic acids, peptide and carbohydrate significantly decreased after 8 weeks of SMG (Supplementary Table S1).Overall, these data indicate that long-term SMG significantly alter the intestinal metabolites, which may mainly contribute to disturbance in circulatory metabolism. We then used Random Forest machine learning analysis to determine the most discriminatory metabolites from SMG and control groups. The top 30 different metabolites including but not limited to 19(R)-Hydroxy-prostaglandin E2, DL-Tryptophan, 2,6-Dihydroxybenzoic acid, Tetrahydroaldosterone, Suberic acid, N-Carbamyl-L-glutamic acid, 5-Hydroxyindole, Prostaglandin D3, and 11-keto Testosterone (CRM), which were primarily from the amino acid, lipid, and organic acids (Figure 4D).

**Figure 5:**
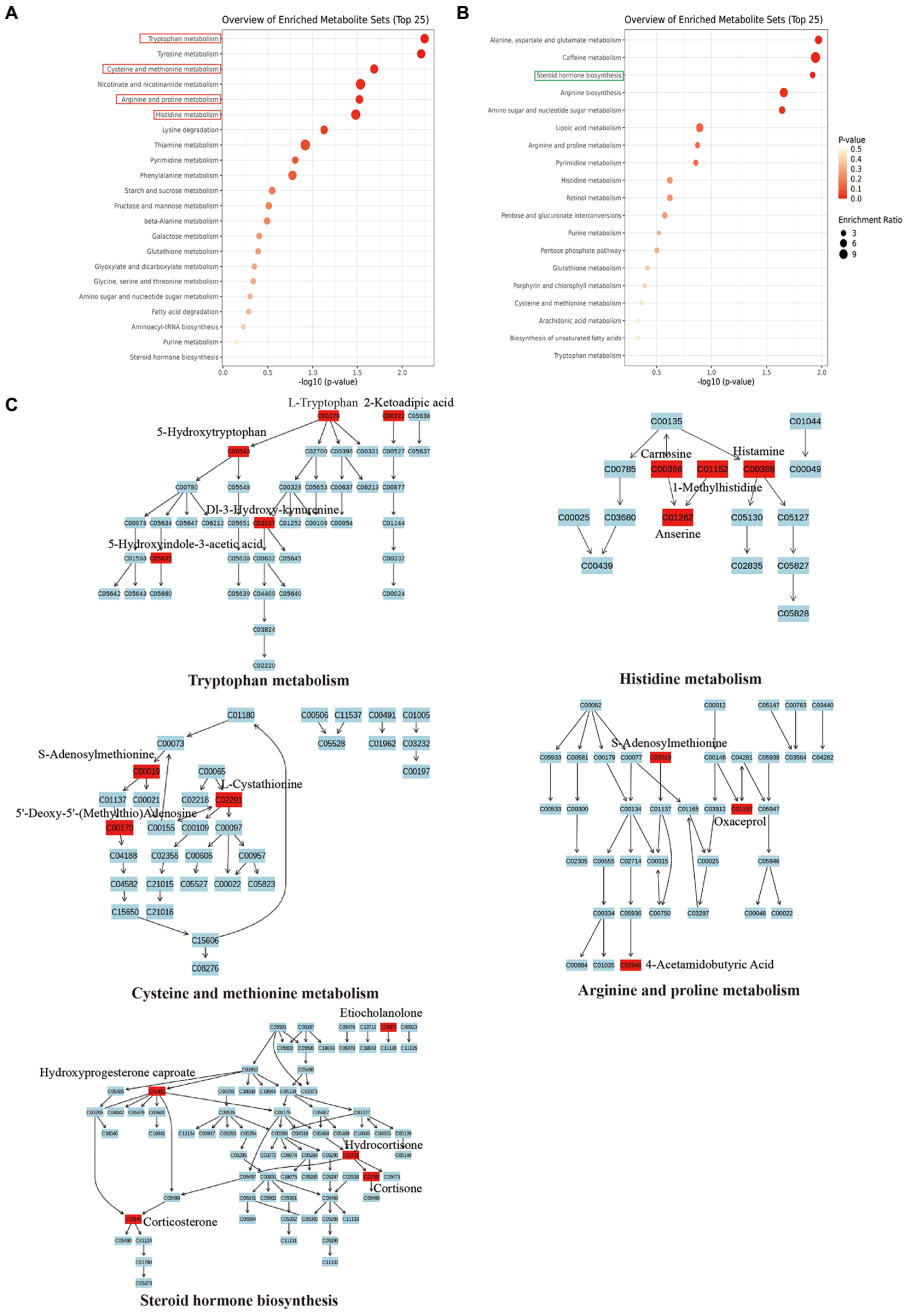
Major metabolic pathway involved in the differentially enriched metabolites of SMG mice compared to controls. **(A)** The major metabolic pathways involved in increased metabolites. **(B)** The major metabolic pathways involved in decreased metabolites. **(C)** Reaction steps for 5 major metabolic pathways both involved in increased and decreased metabolites.

Further, to investigate the potential role of SMG-related metabolites, we annotated metabolic features by using the Kyoto Encyclopedia of Genes and Genomes (KEGG) database. The SMG mice exhibited distinct patterns of metabolites involved in 36 metabolic pathways, including tryptophan metabolism, Tyrosine metabolism, alanine, aspartate and glutamate metabolism, and caffeine metabolism (Supplementary Table S2). Metabolites that were increased in abundance in SMG reflected pathways mainly involved in tryptophan metabolism (L-tryptophan, 2-ketoadipic acid, and 5-hydroxytryptophan), histidine metabolism (carnosine, histamine and 1-methylhistidine), cysteine and methionine metabolism (S-adenosylmethionine, L-cystathionine and 5′-deoxy-5′(methylthio) adenosine), and arginine and proline metabolism (S-adenosylmethionine, oxaceprol, and 4-acetamidobutyric acid) (*p* < 0.05) (Figures 5A,C). Metabolites whose abundance was decreased in SMG reflected pathways involved in steroid hormone biosynthesis (etiocholanolone, hydrocortisone, and cortisone) (*p* < 0.05) (Figures 5B,C). Overall, these results revealed that long-term SMG significantly affects the intestinal metabolites and metabolic pathways.

### Discriminate the origins of metabolites significantly changed

We further sought to determine the potential origin of the metabolites that were highly correlated with gut bacteria. Total of 106 co-metabolism metabolites, 36 microbiota metabolites, and 21 host metabolites were identified (Supplementary Figure S4A,B). We performed functional enrichment analysis of these metabolites and observed that these metabolites were mainly enriched in steroid hormone biosynthesis, histidine metabolism, tryptophan metabolism, tyrosine metabolism, Galactose metabolism, arginine biosynthesis, arginine and proline metabolism, beta-Alanine metabolism, and glutathione metabolism (Supplementary Figure S4C). We found that metabolites related to 9 pathways were originated from microbiota, including tryptophan metabolism, tyrosine metabolism, Galactose metabolism, arginine and proline metabolism, pyrimidine metabolism, folate biosynthesis, aminobenzoate degradation, and carotenoid biosynthesis. Interestingly, only the pathway of steroid hormone biosynthesis was identified to be related to host metabolism and 27 pathways involved in co-metabolism, such as histidine metabolism, tryptophan metabolism, tyrosine metabolism, arginine biosynthesis, arginine and proline metabolism, beta-alanine metabolism, and glutathione metabolism. Collectively, the differential metabolites we identified here were mostly produced or influenced by the gut microbiota. Our results suggested that the altered intestinal metabolism may have a strong correlation with the changed gut microbiota induced by SMG.

### Associations of differential intestinal metabolites with the major bacterial genera

To further investigate the potential dependencies between the perturbed intestinal metabolites and gut microbiota, the Spearman’s correlation coefficient between differential metabolites and the abundance of differential gut bacteria was determined. Strong connections were identified between the perturbed intestinal metabolites and gut microbiota (Table 1). Our results showed that the level of several differential metabolites were significantly correlated with the relative abundance of biomarker microbial genera (ρ > 0.6, *p* < 0.05). Notably, *Allobaculum*, *Akkermansia*, *Phascolarctobacterium*, and *Faecalibacterium* who have been reported to produce SCFAs, exhibited a negative association with metabolites involved in tryptophan metabolism (5-hydroxyindole-3-acetic acid, 2-ketoadipic acid, Dl-3-hydroxy-kynurenine, L-tryptophan, and oxoadipic Acid), histidine metabolism (1-methylhistidine, carnosine, anserine, and histamine), arginine and proline metabolism (S-adenosylmethionine, oxaceprol, and 4-acetamidobutyric Acid), but positive with hydroxyprogesterone caproate and etiocholanolone involving in steroid hormone biosynthesis. Moreover, genera of *Bacteroides, Lachnospiraceae_Clostridium, Rothia, Bilophila,* and *Coprococcus* were positively correlated with arginine and proline metabolism (4-acetamidobutyric acid and oxaceprol), tryptophan metabolism (5-hydroxyindole-3-acetic acid), Dl-3-hydroxy-kynurenine, L-tryptophan), and histidine metabolism (anserine, carnosine, and histamine), but negatively associated with steroid hormone biosynthesis (etiocholanolone, hydroxyprogesterone caproate, and cortisol). The relative abundance of *Alistipes* showed significant positive correlation with histidine metabolism (urocanic acid) and steroid hormone biosynthesis (etiocholanolone and cortisol). Our results suggested that the alteration of intestinal metabolites and pathways under long-term of SMG have a strong association with the imbalance of gut microbiota.

**Table 1:** The Spearman’s correlation significantly changed between intestinal differential metabolites and bacteria genera.

Microbiota	Metabolites	Correlation	*p*-value	*q*-value
*Bacteroides*	Urocanic acid	−0.87413	0.000309	**0.00618**
	5-Hydroxyindole-3-acetic acid	0.839161	0.001192	**0.01192**
	Etiocholanolone	−0.76224	0.005897	**0.03932**
	5α-Dihydrotestosterone	0.727273	0.010001	0.050005
	Histamine	0.706294	0.013286	0.051823
	4-Acetamidobutyric Acid	0.678322	0.018825	0.051823
	Dl-3-Hydroxy-kynurenine	0.671329	0.020442	0.051823
	Anserine	0.664336	0.022159	0.051823
	L-Tryptophan	0.657343	0.023981	0.051823
	Carnosine	0.65035	0.025912	0.051823
	Oxaceprol	0.594406	0.045753	0.083187
*Allobaculum*	5-Hydroxyindole-3-acetic acid	−0.81119	0.002369	**0.02746**
	Dl-3-Hydroxy-kynurenine	−0.8042	0.002746	**0.02746**
	5α-Dihydrotestosterone	−0.76224	0.005897	**0.03932**
	Urocanic acid	0.734266	0.009052	**0.04408**
	Carnosine	−0.72028	0.011021	**0.04408**
	Anserine	−0.68531	0.017305	**0.04543**
	5-Hydroxytryptophan	−0.67832	0.018825	**0.04543**
	4-Acetamidobutyric Acid	−0.67832	0.018825	**0.04543**
	Etiocholanolone	0.671329	0.020442	**0.04543**
	Histamine	−0.65035	0.025912	0.051823
	Oxaceprol	−0.63636	0.030114	0.054753
*Akkermansia*	5-Hydroxyindole-3-acetic acid	−0.62937	0.032395	0.279615
	4-Acetamidobutyric Acid	−0.58741	0.048845	0.279615
*Phascolarctobacterium*	Histamine	−0.73677	0.00627	0.052918
	4-Acetamidobutyric Acid	−0.73321	0.006661	0.052918
	Dl-3-Hydroxy-kynurenine	−0.69406	0.012279	0.052918
	1-Methylhistidine	−0.67982	0.015006	0.052918
	5-Hydroxyindole-3-acetic acid	−0.65847	0.019899	0.052918
	Urocanic acid	0.64423	0.023751	0.052918
	Carnosine	−0.63355	0.026975	0.052918
	S-Adenosylmethionine	−0.62999	0.028117	0.052918
	L-Tryptophan	−0.62643	0.029293	0.052918
	Oxoadipic Acid	−0.62287	0.030504	0.052918
	Hydroxyprogesterone caproate	0.619315	0.031751	0.052918
	Anserine	−0.61932	0.031751	0.052918
	2-Ketoadipic acid	−0.59796	0.040012	0.061557
	5α-Dihydrotestosterone	−0.5766	0.049696	0.070994
*Alistipes*	Urocanic acid	0.79021	0.003617	0.072336
	Etiocholanolone	0.727273	0.010001	0.100009
	Cortisol	0.601399	0.042807	0.285379
*Rothia*	Cortisol	−0.74861	0.005091	0.101826
	Urocanic acid	−0.68622	0.01373	0.101971
	5-Hydroxyindole-3-acetic acid	0.678426	0.015296	0.101971
	5α-Dihydrotestosterone	0.655032	0.020783	0.103917
	Etiocholanolone	−0.60824	0.035863	0.120808
	Oxaceprol	0.600446	0.038979	0.120808
	Dl-3-Hydroxy-kynurenine	0.592648	0.042283	0.120808
*Lachnospiraceae_Clostridium*	Oxaceprol	0.733212	0.006661	0.133225
	5-Hydroxytryptophan	0.633552	0.026975	0.146464
	5-Hydroxyindole-3-acetic acid	0.629993	0.028117	0.146464
	5α-Dihydrotestosterone	0.626434	0.029293	0.146464
	Hydroxyprogesterone caproate	−0.60152	0.038539	0.154158
*Faecalibacterium*	Urocanic acid	0.725045	0.007629	0.152575
	Histamine	−0.6655	0.018175	0.181751
*Coprococcus*	Oxaceprol	0.713287	0.012114	0.239811
	5-Hydroxytryptophan	0.657343	0.023981	0.239811
	5α-Dihydrotestosterone	0.594406	0.045753	0.264343
*Bilophila*	5α-Dihydrotestosterone	0.615385	0.037335	0.312029
	Oxaceprol	0.601399	0.042807	0.312029
*Rikenella*	Cortisol	−0.64086	0.024736	0.369857
*Anaerotruncus*	Oxaceprol	0.65035	0.025912	0.43853

In table, q value less than 0.05 indicantes a significant correlation with the responding genus, which is highlighted in bold.

## Discussion

Accumulating evidence showed that microgravity affects bone, muscle and the gut microbiota, yet the effect of long-term microgravity on intestinal microbiome and metabolism remains unclear. Hence, we dynamically compared the intestinal microbiome and metabolome between SMG and control mice during the period of eight weeks, and we found that SMG induced no significant difference in diversity and richness of intestinal flora, but altered the composition of the intestinal microbiome at week 2, 4 and 8. This observation was inconsistence with an earlier work, which reported that 3 weeks of SMG reduce the diversity of colonic microbiota (Wang et al., 2020). These results indicated that SMG have distinct effects on bacterial flora in different period of time. Meanwhile, the intestinal metabolites and metabolic pathways changed characteristically under long-term SMG. Together, the current study contributes to identifying microbes, metabolites, and metabolic pathways that are mostly affected by a long-term SMG, and may be associated with the pathogenesis of SMG-associated diseases.

In line with previous reports, this research suggested that SMG altered the structural composition of gut microbiota. A previous study found that 3 weeks of SMG increased the relative abundance of *Proteobacteria* (Wang et al., 2020). However, in our study, the phylum of *Verrucomicrobia* significantly declined from weeks 2 to 8, yet the relative abundance of *Actinobacteria* and *Deferribacteres* raised remarkably at week 8. These findings indicated that the influence of SMG on gut microbiota is different across different time intervals. Moreover, at the genus level, *Allobaculum* and *Bacteroides* showed the most remarkable discrepancy between SMG and control groups during the whole 8 weeks period. *Allobaculum* is a kind of glucose utilizers and producers of lactate and butyrate, and its decline has been reported to be correlated with cognitive impairments, aging, high-fat diets, inflammatory disease and fatty acid metabolism (Bárcena et al., 2019; Pujo et al., 2021; Hu et al., 2022; Wang et al., 2022). While, the genus *Bacteroides* considered as friendly commensal bacteria, overgrowth of which may produce different disease conditions by competed nutrition with other gut commensals or transfer virulence genes, was enriched in the SMG group (Zafar and Saier, 2021). Further, Cambell et al. have reported increased levels of *Bacteroides* spp. in cancer patients (Parida and Sharma, 2019). In another study, they have reported that *Bacteroides* spp. may trigger autoimmunity accompanied by cardiomyopathy (Gil-Cruz et al., 2019). Hence, this study identifies *Allobaculum* and *Bacteroides* as biomarker bacteria mostly affected by longer-term microgravity in a simulated microgravity mouse model.

We also found that SMG continuously decreased several beneficial bacterial species such as *Bacteroides_uniformis, Akkermansia_muciniphila,* and *Faecalibacterium_prausnitzii* at different time points. A previous study reported that supplementation with *Bacteroides_uniformis* could improve ASD-like behaviors by decreasing serum glutamine levels and intestinal amino acid transport (Yu et al., 2022). In addition, *Akkermansia_muciniphila* is a well-known mucin-degrading bacterium, playing a critical role in the thickening of mucus layers and intestinal barrier improvement. Loss of *Akkermansia_muciniphila* correlated with inflammation, impairment of insulin secretion and glucose homeostasis, metabolic disease, cancer immunotherapy, and homeostatic immunity (Yoon et al., 2021; Zhang et al., 2021; Bae et al., 2022; Schneider et al., 2022). *Faecalibacterium_prausnitzii*, an important butyrate-producer of the phylum of *Firmicutes*, has been suggested as a biosensor and a major player of the intestinal health. The reduction of *Faecalibacterium_prausnitzii* has been reported to be associated with inflammation, metabolic disease and type-2 diabetes (Verhoog et al., 2019). Interestingly, two potential pathogenic bacterial species *Ruminococcus gnavus* and *Mucispirillum schaedleri* were increased significantly at week 8 of SMG. Multiple studies associate the mucin-glycan foraging *Ruminococcus gnavus* with Crohn’s disease, IBD, allergic disease and neurological disorder (Hall et al., 2017; Chua et al., 2018; Henke et al., 2019; Coletto et al., 2022). Recently, *Mucispirillum schaedleri*, belonging to the phylum *Deferribacteres*, was also linked to inflammatory conditions in the intestine (Herp et al., 2021). Taken together, our results verify the gut bacterial biomarkers dynamically caused by SMG, probably leading to microgravity-associated systemic imbalance.

Changes in the gut microbiota have been closely linked to intestinal and host metabolism. We firstly performed a comprehensive profiling of the fecal metabolites from control and SMG mice at week 8. Our results suggested that specific individual metabolites and metabolic pathways were significantly altered, involving in tryptophan metabolism, histidine metabolism, cysteine and methionine metabolism, arginine and proline metabolism, and **s**teroid hormone biosynthesis, which show a strong association with different microbes. Next, we applied a MetOrigin database to distinguish origination of metabolites, and our results verified that the disturbed metabolites mostly resulted from co-metabolism or originated from microbiota. In the last few decades, role of the gut microbiota has been suggested in many diseases, such as IBD, metabolic syndrome and associated complications, and neuropsychiatric disorders which is partially mediated by impaired Trp metabolism (Agus et al., 2018). Previous literature depicts that gut microbiota altered proline metabolism in depression (Mayneris-Perxachs et al., 2022), implying its essential role in depression by regulating metabolism. Gut microbes also impacts intestinal motility through histamine secretion and activation of histamine receptors, and bacterial histidine decarboxylases are enriched in patients with Crohn’s disease (Chen et al., 2019). Histidine and arginine metabolism have been proven as potential immunomodulatory pathways mediated by *Akkermansia muciniphila* and *Bifidobacterium longum* (Stražar et al., 2021). In this study, *Akkermansia muciniphila* and some other specific bacteria showed a negative correlation with the metabolites involved in histidine, arginine and proline metabolism, indicating that long-term SMG-induced intestinal microbiota imbalance may affect host inflammatory response and neuropsychiatric disorders by regulating intestinal metabolism.

Gut microbiota dysbiosis affects steroid hormone metabolism contributing to several diseases, such as cancer development, hypertension, obesity, and neurological disorders (Daisley et al., 2020; Yan et al., 2020; Mayneris-Perxachs et al., 2020a; Li et al., 2022). Yan et.al showed that intestinal microbiota modulated blood pressure by affecting corticosterone level (Yan et al., 2020). Gut microbiota participate in **s**teroid hormone biosynthesis and degradation. The gut microbiota of pre-menopausal women enriched in genes from the steroid biosynthesis and degradation, and women harbor microbial community type with lower abundance of *Ruminococcaceae*, *Faecalibacterium*, and *Alistipes*, but higher abundance of *Bacteroides* and *Prevotella* in another study (Ding and Schloss, 2014; Mayneris-Perxachs et al., 2020b). Here, our results demonstrated that intestinal metabolites involved in **s**teroid hormone biosynthesis were significantly reduced by 8 weeks of SMG, and strongly associated with special microbes, such as *Bacteroides*, *Alistipe*s, *Allobaculum*, *Faecalibacterium,* and *Phascolarctobacterium*. Collectively, these results indicated that long-term SMG-induced gut microbiota leads to intestinal metabolic disturbance directly or indirectly, which may affect host health. Furthermore, systematic research is required to explore the potential role of altered intestinal flora in microgravity-associated host dysfunction.

## Conclusion

To the best of our knowledge, this is the first research of integrated intestinal microbiome and metabolomics in the simulated microgravity mice model under longer time of SMG. Different gut microbial biomarkers were verified at different time points of SMG. *Allobaculum* and *Bacteroide*s were identified as biomarkers for the whole process of SMG. *Bacteroides_uniformis*, *Faecalibacterium_prausnitzii*, *Ruminococcus_flavefaciens,* and *Mucispirillum_schaedleri* were most affected species under 8 weeks of SMG. This long-term SMG-induced intestinal dysbiosis has a strong association with differential gut metabolites participating in metabolic pathways including tryptophan metabolism, histidine metabolism, arginine and proline metabolism, and steroid hormone biosynthesis. Our results indicated that long-term SMG has a more significant impact on intestinal microbiota, which further influence the intestinal metabolism. Our work will provide a better understanding of the long-term microgravity impact on intestinal homeostasis, and urges further deep explorations of the relationships between gut microbial dysbiosis, metabolism and microgravity-induced host dysfunction.

## Data availability statement

The datasets presented in this study can be found in online repositories. The names of the repository/repositories and accession number(s) can be found below: MTBLS6526 PRJNA905934.

## Ethics statement

The animal study was reviewed and approved by the Institutional Experimental Animal Care and Use Committee of the PLA Strategic Support Force Medical Center.

## Author contributions

LY and RZ designed the research and drafted the manuscript. XL and SS co-supervised the project and revised the manuscript. LY, RZ, XM, LZ, QP, and MW performed the experiments. LX, CG, and XH helped analyzing the data. SH revised the manuscript. All authors read and approved the final manuscript.

## Funding

This work was supported by the scientific research fund project for doctoral graduates by the PLA Strategic Support Force Medical Center (21ZXO8).

## Conflict of interest

The authors declare that the research was conducted in the absence of any commercial or financial relationships that could be construed as a potential conflict of interest.

## Publisher’s note

All claims expressed in this article are solely those of the authors and do not necessarily represent those of their affiliated organizations, or those of the publisher, the editors and the reviewers. Any product that may be evaluated in this article, or claim that may be made by its manufacturer, is not guaranteed or endorsed by the publisher.

## Footnotes

^1^ https://docs.qiime2.org/2019.1/

^2^ https://www.mzcloud.org/

^3^ http://metorigin.met-bioinformatics.cn/

^4^ https://cloud.oebiotech.com/task/

^5^ https://www.metaboanalyst.ca/
