# Supplementary figures and images for: Long-term simulated microgravity alters gut microbiota and metabolome in mice
Source: Front Microbiol. 2023 Mar 24;14:1100747. doi: 10.3389/fmicb.2023.1100747 (PMC10080065; doi:10.3389/fmicb.2023.1100747)

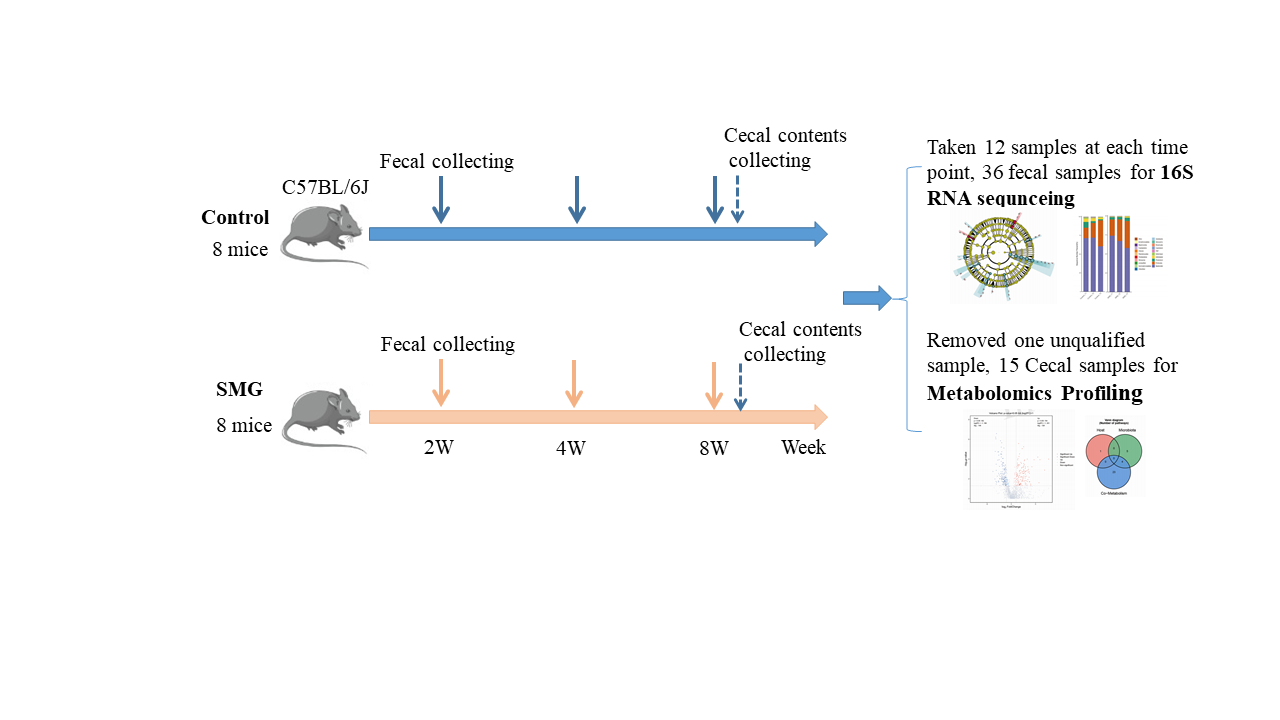

Supplement: Supplementary file 1 [file Image_1.TIF]

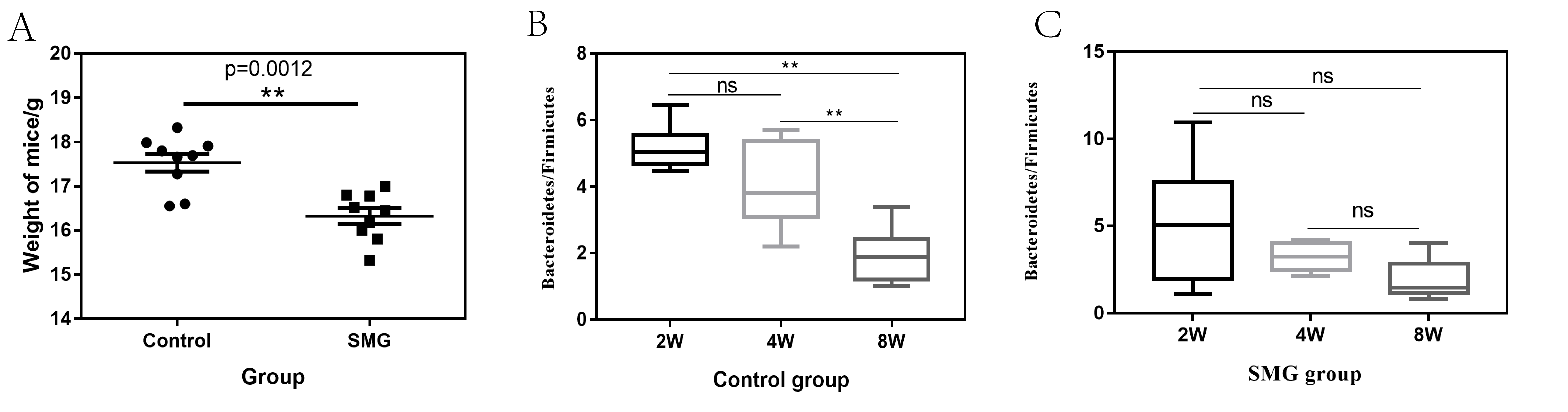

Supplement: Supplementary file 2 [file Image_2.TIF]

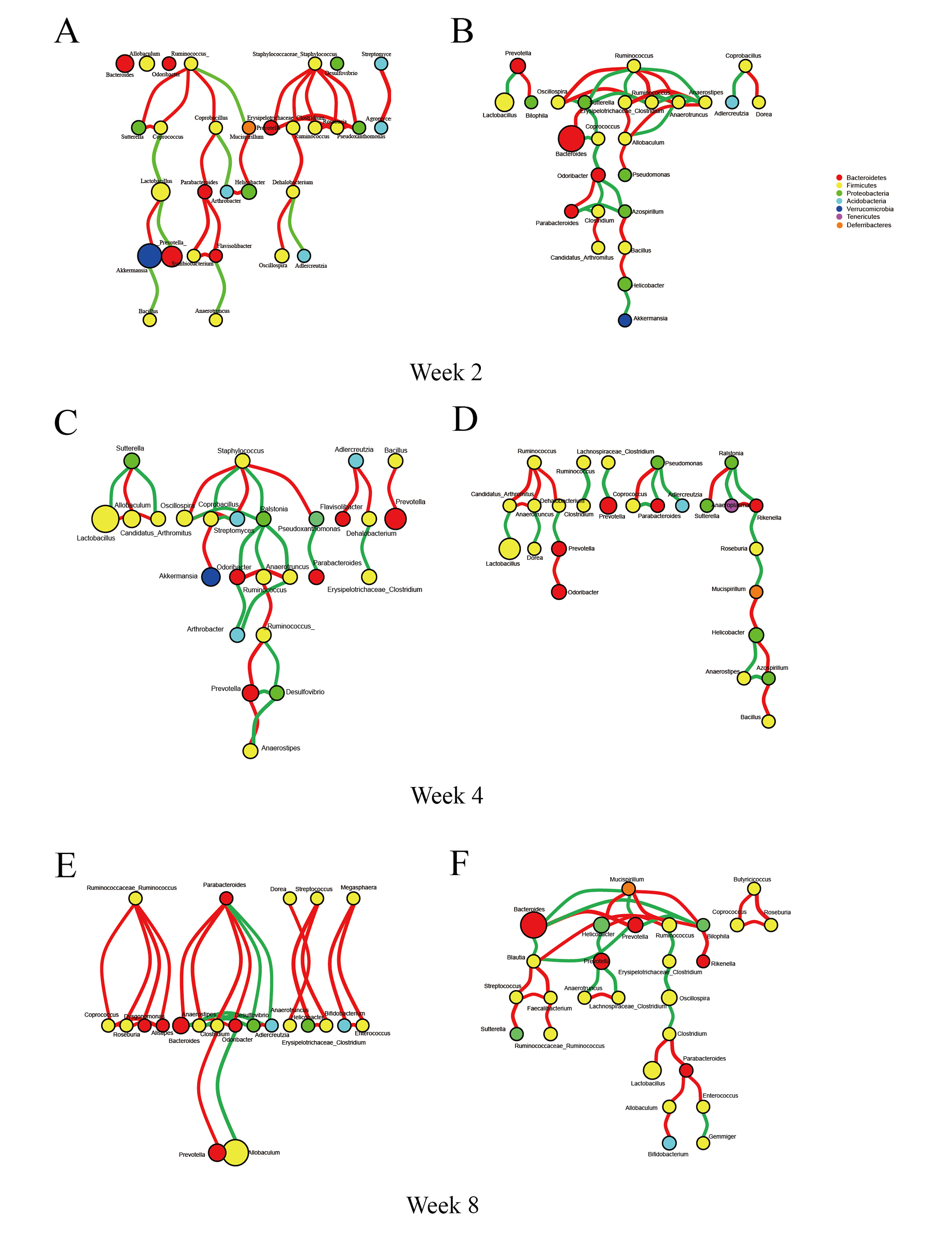

Supplement: Supplementary file 3 [file Image_3.TIF]

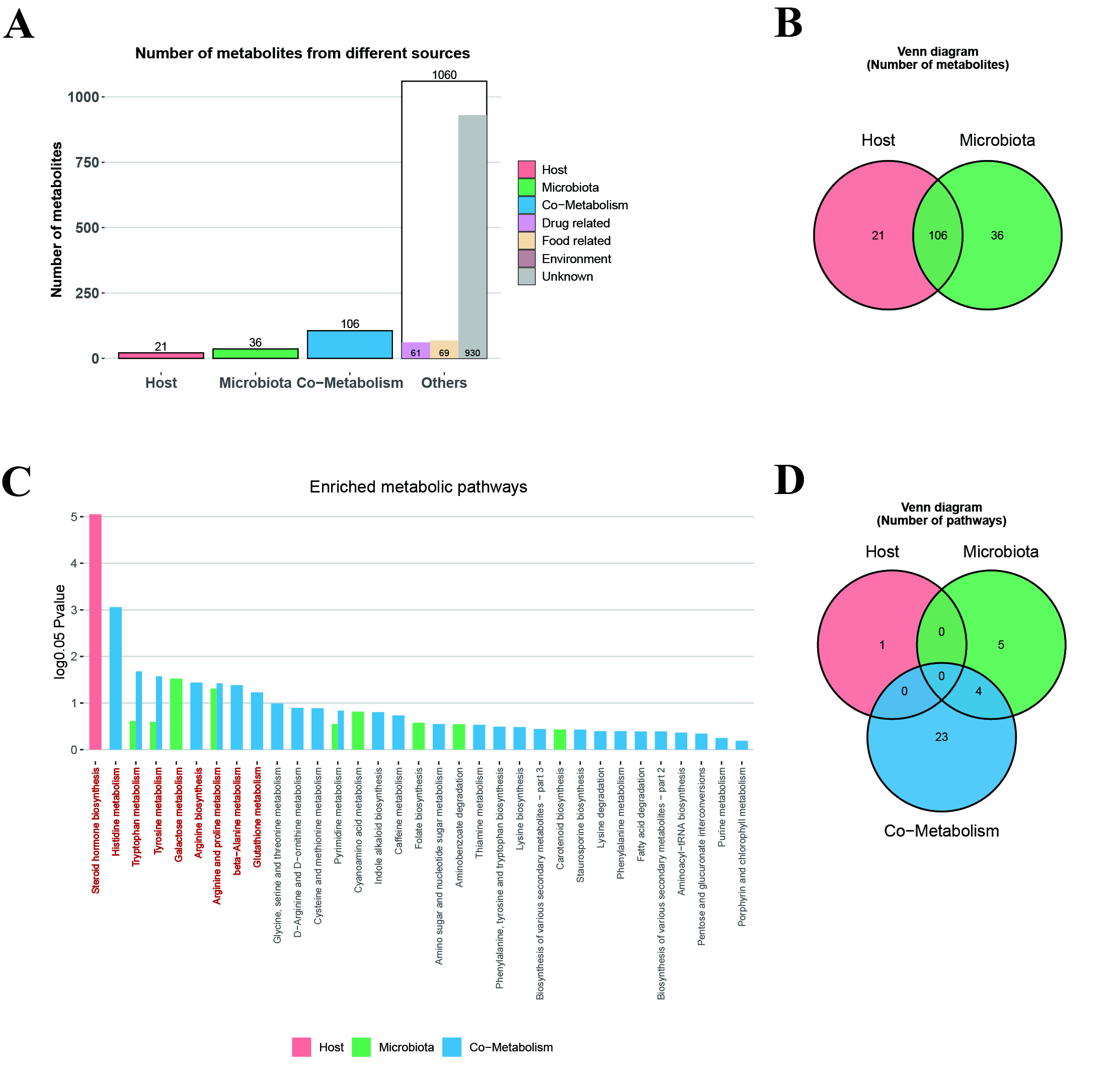

Supplement: Supplementary file 4 [file Image_4.TIF]
