# Supplementary material for: Long-term simulated microgravity alters gut microbiota and metabolome in mice
Source: Front Microbiol. 2023 Mar 24;14:1100747. doi: 10.3389/fmicb.2023.1100747 (PMC10080065; doi:10.3389/fmicb.2023.1100747)
Supplement: Supplementary file 5 [file Table_1.DOCX]

**Table S1** The relative abundance of metabolites on the super class level

| Class | Group | | P value |
| --- | --- | --- | --- |
|  | Control | SMG |  |
| Steroids | 0.5762±0.1515 | 0.6484±0.2650 | 0.152 |
| Lipids | 0.2840±0.1379 | 0.2288±0.2269 | 0.232 |
| Vitamins and Cofactors | 0.0527±0.0082 | 0.0608±0.0231 | 1.000 |
| **Organic acids** | 0.0318±0.0084 | 0.0125±0.0250 | **0.040^a^** |
| **Peptides** | 0.0228±0.0049 | 0.0168±0.0114 | **0.040^a^** |
| Nucleic acids | 0.0150±0.0043 | 0.0145±0.0065 | 0.536 |
| **Carbohydrates** | 0.0145±0.0042 | 0.0079±0.0034 | **0.006^b^** |
| Hormones and transmitters | 0.0020±0.0004 | 0.0023±0.0012 | 1.000 |
| Antibiotics | 0.0009±0.0002 | 0.0014±0.0008 | 0.536 |

The relative abundance of metabolites is presented as mean ± SD. The letter a represents p˂0.05, b represents p˂0.01.
