# Supplementary material for: Long-term simulated microgravity alters gut microbiota and metabolome in mice
Source: Front Microbiol. 2023 Mar 24;14:1100747. doi: 10.3389/fmicb.2023.1100747 (PMC10080065; doi:10.3389/fmicb.2023.1100747)
Supplement: Supplementary file 6 [file Table_2.DOCX]

**Table S2** Significantly different metabolic pathways between two groups

| Unregulated pathways | total | hits | p-value | Downregulated pathways | total | hits | p-value |
| --- | --- | --- | --- | --- | --- | --- | --- |
| **Tryptophan metabolism** | 41 | 4 | **0.00563** | **Alanine, aspartate and glutamate metabolism** | 28 | 3 | **0.0107** |
| **Tyrosine metabolism** | 42 | 4 | **0.00614** | **Caffeine metabolism** | 10 | 2 | **0.0114** |
| **Cysteine and methionine metabolism** | 33 | 3 | **0.0205** | **Steroid hormone biosynthesis** | 85 | 5 | **0.0121** |
| **Nicotinate and nicotinamide metabolism** | 15 | 2 | **0.0291** | **Arginine biosynthesis** | 14 | 2 | **0.0221** |
| **Arginine and proline metabolism** | 38 | 3 | **0.0299** | **Amino sugar and nucleotide sugar metabolism** | 37 | 3 | **0.0229** |
| **Histidine metabolism** | 16 | 2 | **0.0329** | Lipoic acid metabolism | 8 | 1 | 0.128 |
| Lysine degradation | 25 | 2 | 0.0743 | Arginine and proline metabolism | 38 | 2 | 0.134 |
| Thiamine metabolism | 7 | 1 | 0.121 | Pyrimidine metabolism | 39 | 2 | 0.139 |
| Pyrimidine metabolism | 39 | 2 | 0.157 | Histidine metabolism | 16 | 1 | 0.24 |
| Phenylalanine metabolism | 10 | 1 | 0.168 | Retinol metabolism | 16 | 1 | 0.24 |
| Starch and sucrose metabolism | 18 | 1 | 0.283 | Pentose and glucuronate interconversions | 18 | 1 | 0.266 |
| Fructose and mannose metabolism | 20 | 1 | 0.309 | Purine metabolism | 65 | 2 | 0.302 |
| beta-Alanine metabolism | 21 | 1 | 0.322 | Pentose phosphate pathway | 22 | 1 | 0.315 |
| Galactose metabolism | 27 | 1 | 0.394 | Glutathione metabolism | 28 | 1 | 0.383 |
| Glutathione metabolism | 28 | 1 | 0.405 | Porphyrin and chlorophyll metabolism | 30 | 1 | 0.404 |
| Glyoxylate and dicarboxylate metabolism | 32 | 1 | 0.448 | Cysteine and methionine metabolism | 33 | 1 | 0.434 |
| Glycine, serine and threonine metabolism | 33 | 1 | 0.459 | Arachidonic acid metabolism | 36 | 1 | 0.463 |
| Amino sugar and nucleotide sugar metabolism | 37 | 1 | 0.498 | Biosynthesis of unsaturated fatty acids | 36 | 1 | 0.463 |
| Fatty acid degradation | 39 | 1 | 0.516 | Tryptophan metabolism | 41 | 1 | 0.508 |
| Aminoacyl-tRNA biosynthesis | 48 | 1 | 0.592 |  |  |  |  |
| Purine metabolism | 65 | 1 | 0.705 |  |  |  |  |
| Steroid hormone biosynthesis | 85 | 1 | 0.8 |  |  |  |  |
