# Supplementary material for: Long-term simulated microgravity alters gut microbiota and metabolome in mice
Source: Front Microbiol. 2023 Mar 24;14:1100747. doi: 10.3389/fmicb.2023.1100747 (PMC10080065; doi:10.3389/fmicb.2023.1100747)
Supplement: Supplementary file 7 [file Table_3.DOCX]

**Table S3** Identification of the origin of metabolites identified by LC/MS

| Metabolites name | HMDBID | KEGGID | Origin |
| --- | --- | --- | --- |
| Etiocholanolone | HMDB0000490 | C04373 | Host |
| Cortisol | HMDB0000063 | C00735 | Host |
| Corticosterone | HMDB0001547 | C02140 | Host |
| Hydrocortisone | HMDB0000063 | C00735 | Host |
| Cortisone | HMDB0002802 | C00762 | Host |
| Progesterone | HMDB0001830 | C00410 | Host |
| 2-Methoxyestrone | HMDB0000010 | C05299 | Host |
| Aldosterone | HMDB0000037 | C01780 | Host |
| Adrenosterone | HMDB0006772 | C05285 | Host |
| Pregnenolone | HMDB0000253 | C01953 | Host |
| Desoxycortone | HMDB0000016 | C03205 | Host |
| Phenylacetylglycine | HMDB0000821 | C05598 | Host |
| Morphine | HMDB0014440 | C01516 | Host |
| Cortodoxone | HMDB0000015 | C05488 | Host |
| (9cis)-Retinal | HMDB0006218 | C16681 | Host |
| Androsterone | HMDB0000031 | C00523 | Host |
| NNK | HMDB0011603 | C16453 | Host |
| All-Trans-13,14-Dihydroretinol | HMDB0011618 | C15492 | Host |
| Tetrahydrocorticosterone | HMDB0000268 | C05476 | Host |
| 13-HPODE | HMDB0003871 | C04717 | Host |
| 3-Hydroxylidocaine | HMDB0060655 | C16560 | Host |
| Phloretin | HMDB0003306 | C00774 | Microbiota |
| Creatinine | HMDB0000562 | C00791 | Microbiota |
| 3-Methylindole | HMDB0000466 | C08313 | Microbiota |
| Astaxanthin | HMDB0002204 | C08580 | Microbiota |
| (S)-Equol | HMDB0002209 | C14131 | Microbiota |
| Biopterin | HMDB0000468 | C06313 | Microbiota |
| Catechol | HMDB0000957 | C15571 | Microbiota |
| (R)-Prunasin | HMDB0034934 | C00844 | Microbiota |
| Tyrosol | HMDB0004284 | C06044 | Microbiota |
| D-Galactosamine | NA | C02262 | Microbiota |
| Pyrogallol | HMDB0013674 | C01108 | Microbiota |
| N-Methylhydantoin | HMDB0003646 | C02565 | Microbiota |
| N-Acetyl-D-galactosamine | HMDB0000212 | C01132 | Microbiota |
| Cytosine | HMDB0000630 | C00380 | Microbiota |
| Maltotetraose | HMDB0001296 | C02052 | Microbiota |
| delta-Tocopherol | HMDB0002902 | C14151 | Microbiota |
| 3-Indoxylsulphate | HMDB0000682 | NA | Microbiota |
| Oxytetracycline | HMDB0014733 | C06624 | Microbiota |
| N-Acetylphenylalanine | HMDB0000512 | C03519 | Microbiota |
| N6-Acetyl-L-lysine | HMDB0000206 | C02727 | Microbiota |
| Ergothioneine | HMDB0003045 | C05570 | Microbiota |
| L-Ergothioneine | HMDB0003045 | C05570 | Microbiota |
| Boldione | HMDB0003422 | C20144 | Microbiota |
| Hydroquinone | HMDB0002434 | C00530 | Microbiota |
| Caffeicacid | HMDB0001964 | C01481 | Microbiota |
| Stercobilin | HMDB0240259 | C05793 | Microbiota |
| 4-Methylphenol | HMDB0001858 | C01468 | Microbiota |
| gamma-Tocopherol | HMDB0001492 | C02483 | Microbiota |
| IPH | HMDB0000228 | C15584 | Microbiota |
| N-(5-Aminopentyl)acetamide | HMDB0002284 | NA | Microbiota |
| Indole | HMDB0000738 | C00463 | Microbiota |
| D-(-)-Mannitol | HMDB0000765 | C00392 | Microbiota |
| D-Alanyl-D-alanine | HMDB0003459 | C00993 | Microbiota |
| 2-Isopropylmalate | HMDB0000402 | C02504 | Microbiota |
| Styrene | HMDB0034240 | C07083 | Microbiota |
| o-Cresol | HMDB0002055 | C01542 | Microbiota |
| Biliverdin | HMDB0001008 | C00500 | Co-Metabolism |
| S-Adenosyl-L-methionine | HMDB0001185 | C00019 | Co-Metabolism |
| Noradrenaline | HMDB0000216 | C00547 | Co-Metabolism |
| D-(-)-Fructose | HMDB0000660 | C02336 | Co-Metabolism |
| 1-Methylhistidine | HMDB0000001 | C01152 | Co-Metabolism |
| Oxaceprol | HMDB0000725 | C01157 | Co-Metabolism |
| Xylitol | HMDB0002917 | C00379 | Co-Metabolism |
| Carnosine | HMDB0000033 | C00386 | Co-Metabolism |
| Guanine | HMDB0000132 | C00242 | Co-Metabolism |
| Histamine | HMDB0000870 | C00388 | Co-Metabolism |
| S-Adenosylmethionine | HMDB0001185 | C00019 | Co-Metabolism |
| L-Ornithine | HMDB0000214 | C00077 | Co-Metabolism |
| Anserine | HMDB0000194 | C01262 | Co-Metabolism |
| Deoxycytidine | HMDB0000014 | C00881 | Co-Metabolism |
| Cytidine | HMDB0000089 | C00475 | Co-Metabolism |
| 2-Phenylethylamine | HMDB0012275 | C05332 | Co-Metabolism |
| Metanephrine | HMDB0004063 | C05588 | Co-Metabolism |
| N-acetyl-D-glucosamine | HMDB0000215 | C00140 | Co-Metabolism |
| Citrulline | HMDB0000904 | C00327 | Co-Metabolism |
| L-Saccharopine | HMDB0000279 | C00449 | Co-Metabolism |
| gamma-Glutamylcysteine | HMDB0001049 | C00669 | Co-Metabolism |
| 5,6-dihydroxyindole | HMDB0004058 | C05578 | Co-Metabolism |
| 5-Hydroxytryptophan | HMDB0000472 | C00643 | Co-Metabolism |
| Thiamine | HMDB0000235 | C00378 | Co-Metabolism |
| Palmitoylcarnitine | HMDB0000222 | C02990 | Co-Metabolism |
| DL-Citrulline | HMDB0000904 | C00327 | Co-Metabolism |
| 1-Methylxanthine | HMDB0010738 | C16358 | Co-Metabolism |
| 6-Hydroxymelatonin | HMDB0004081 | C05643 | Co-Metabolism |
| L-Tryptophan | HMDB0000929 | C00078 | Co-Metabolism |
| L-Cystathionine | HMDB0000099 | C02291 | Co-Metabolism |
| Levodopa | HMDB0000181 | C00355 | Co-Metabolism |
| Adenine | HMDB0000034 | C00147 | Co-Metabolism |
| Tretinoin | HMDB0001852 | C00777 | Co-Metabolism |
| L-Dopa | HMDB0000181 | C00355 | Co-Metabolism |
| dopaquinone | HMDB0001229 | C00822 | Co-Metabolism |
| L-Malate | HMDB0000156 | C00149 | Co-Metabolism |
| Cholesterol | HMDB0000067 | C00187 | Co-Metabolism |
| L-lysine | HMDB0000182 | C00047 | Co-Metabolism |
| Estriol | HMDB0000153 | C05141 | Co-Metabolism |
| N-Formylkynurenine | HMDB0001200 | C02700 | Co-Metabolism |
| Tyramine | HMDB0000306 | C00483 | Co-Metabolism |
| 7-Methylxanthine | HMDB0001991 | C16353 | Co-Metabolism |
| L-Serine | HMDB0000187 | C00065 | Co-Metabolism |
| D-(+)-Xylose | HMDB0000098 | C00181 | Co-Metabolism |
| Phenylacetaldehyde | HMDB0006236 | C00601 | Co-Metabolism |
| N-Acetylmannosamine | HMDB0001129 | C00645 | Co-Metabolism |
| Pantetheine | HMDB0003426 | C00831 | Co-Metabolism |
| L-Pipecolate | HMDB0000070 | C00408 | Co-Metabolism |
| Bilirubin | HMDB0000054 | C00486 | Co-Metabolism |
| N-Acetylornithine | HMDB0003357 | C00437 | Co-Metabolism |
| Uridine | HMDB0000296 | C00299 | Co-Metabolism |
| Choline | HMDB0000097 | C00114 | Co-Metabolism |
| 5beta-Androstane-3,17-dione | HMDB0003769 | C03772 | Co-Metabolism |
| Thymidine | HMDB0000273 | C00214 | Co-Metabolism |
| Xanthosine | HMDB0000299 | C01762 | Co-Metabolism |
| Porphobilinogen | HMDB0000245 | C00931 | Co-Metabolism |
| L-Histidine | HMDB0000177 | C00135 | Co-Metabolism |
| O-Phospho-L-serine | HMDB0000272 | C01005 | Co-Metabolism |
| Threonine | HMDB0000167 | C00188 | Co-Metabolism |
| D-Proline | HMDB0003411 | C00763 | Co-Metabolism |
| Testosterone | HMDB0000234 | C00535 | Co-Metabolism |
| 17-Hydroxyprogesterone | HMDB0000374 | C01176 | Co-Metabolism |
| L-Phenylalanine | HMDB0000159 | C00079 | Co-Metabolism |
| L-Threonine | HMDB0000167 | C00188 | Co-Metabolism |
| Creatine | HMDB0000064 | C00300 | Co-Metabolism |
| Methylmalonate | HMDB0000202 | C02170 | Co-Metabolism |
| Glutathione | HMDB0000125 | C00051 | Co-Metabolism |
| 5-oxoproline | HMDB0000267 | C01879 | Co-Metabolism |
| Riboflavin | HMDB0000244 | C00255 | Co-Metabolism |
| L-Kynurenine | HMDB0000684 | C00328 | Co-Metabolism |
| acetoacetate | HMDB0000060 | C00164 | Co-Metabolism |
| Xanthine | HMDB0000292 | C00385 | Co-Metabolism |
| Glucose-6-phosphate | HMDB0001401 | C00092 | Co-Metabolism |
| Phosphocholine | HMDB0001565 | C00588 | Co-Metabolism |
| Biotin | HMDB0000030 | C00120 | Co-Metabolism |
| SPH | HMDB0000252 | C00319 | Co-Metabolism |
| D-Sphingosine | HMDB0000252 | C00319 | Co-Metabolism |
| 2-Deoxyadenosine | HMDB0000101 | C00559 | Co-Metabolism |
| 3-Methoxytyramine | HMDB0000022 | C05587 | Co-Metabolism |
| L-Asparagine | HMDB0000168 | C00152 | Co-Metabolism |
| Estrone | HMDB0000145 | C00468 | Co-Metabolism |
| L-Tyrosine | HMDB0000158 | C00082 | Co-Metabolism |
| D-(+)-Maltose | HMDB0000163 | C00208 | Co-Metabolism |
| Guanosine | HMDB0000133 | C00387 | Co-Metabolism |
| Hypoxanthine | HMDB0000157 | C00262 | Co-Metabolism |
| N6,N6,N6-Trimethyl-L-lysine | HMDB0001325 | C03793 | Co-Metabolism |
| Inosine | HMDB0000195 | C00294 | Co-Metabolism |
| Cholecalciferol | HMDB0000876 | C05443 | Co-Metabolism |
| S-Adenosylhomocysteine | HMDB0000939 | C00021 | Co-Metabolism |
| Thymine | HMDB0000262 | C00178 | Co-Metabolism |
| Sucrose | HMDB0000258 | C00089 | Co-Metabolism |
| DL-3,4-Dihydroxyphenylglycol | HMDB0000318 | C05576 | Co-Metabolism |
| dCMP | HMDB0001202 | C00239 | Co-Metabolism |
| Nicotinamide | HMDB0001406 | C00153 | Co-Metabolism |
| S-Adenosyl-methionine | HMDB0001185 | C00019 | Co-Metabolism |
| GDP | HMDB0001201 | C00035 | Co-Metabolism |
| Adenosine | HMDB0000050 | C00212 | Co-Metabolism |
| D-Raffinose | HMDB0003213 | C00492 | Co-Metabolism |
| cGMP | HMDB0001314 | C00942 | Co-Metabolism |
| L-cysteine | HMDB0000574 | C00097 | Co-Metabolism |
| Deoxyinosine | HMDB0000071 | C05512 | Co-Metabolism |
| L-Argininosuccinate | HMDB0000052 | C03406 | Co-Metabolism |
| Uracil | HMDB0000300 | C00106 | Co-Metabolism |
| Taurine | HMDB0000251 | C00245 | Co-Metabolism |
| Estradiol | HMDB0000151 | C00951 | Co-Metabolism |
| 2-Deoxyuridine | HMDB0000012 | C00526 | Co-Metabolism |
