# Supplementary material for: Long-term simulated microgravity alters gut microbiota and metabolome in mice
Source: Front Microbiol. 2023 Mar 24;14:1100747. doi: 10.3389/fmicb.2023.1100747 (PMC10080065; doi:10.3389/fmicb.2023.1100747)
Supplement: Supplementary file 8 [file Table_4.DOCX]

**Table S4** Significantly different metabolites between control and SMG groups

| Metabolites | Control-Mean | SMG-Mean | p.value |
| --- | --- | --- | --- |
| Paracetamol | 2.859819 | 0.346565 | 2.61E-07 |
| PC (18:4e/18:4) | 0.77366 | 0.083198 | 6.04E-07 |
| 3b,7b-Dihydroxy-5-androsten-17-one | 3.567506 | 0.395041 | 8.71E-07 |
| S-Adenosyl-L-methionine | 0.255875 | 0.054871 | 1.39E-06 |
| 2,3-Dinor-8-epi-prostaglandin F2α | 4.008112 | 1.027389 | 2.21E-06 |
| 3-(3-pyridinyl)propanoic acid | 0.119018 | 0.339775 | 2.65E-06 |
| 2-{[methyl(2,3,4,5,6-pentahydroxyhexyl)amino]methylidene}malononitrile | 0.742538 | 4.349491 | 5.54E-06 |
| Suberic acid | 19.6303 | 4.712432 | 6.15E-06 |
| (+)-Aphidicolin | 1.440726 | 0.502736 | 7.65E-06 |
| 5α-Dihydrotestosterone | 0.255919 | 1.224646 | 8.58E-06 |
| 3-Hydroxysebacic acid | 44.46391 | 4.515318 | 1.68E-05 |
| Prostaglandin D3 | 5.661716 | 1.453224 | 1.78E-05 |
| (-)-Erythromycin | 0.281183 | 0.04516 | 1.80E-05 |
| Lysopg 18:1 | 15.60506 | 2.345633 | 1.89E-05 |
| Linoleoyl ethanolamide | 7.089538 | 23.14994 | 2.97E-05 |
| Tetradecanedioic acid | 12.21185 | 2.948261 | 3.00E-05 |
| Tetrahydroaldosterone | 3.875988 | 0.835677 | 3.11E-05 |
| Biliverdin | 7.811106 | 1.962687 | 3.39E-05 |
| 11-keto Testosterone (CRM) | 6.41597 | 1.0657 | 3.97E-05 |
| 5-Hydroxyindole | 0.168152 | 0.504574 | 5.51E-05 |
| 13,14-dihydro-15-keto-tetranor Prostaglandin D2 | 3.893806 | 0.940571 | 6.03E-05 |
| Pyridoxine O-Glucoside | 0.09129 | 3.99363 | 6.26E-05 |
| Etiocholanolone | 11.50912 | 3.598446 | 8.65E-05 |
| cis-2-Decenoic acid | 1.546632 | 0.447363 | 8.70E-05 |
| 1,1-Dimethyl-2-oxopropyl N-[2-(2-pyridyl)ethyl]carbamate | 1.828754 | 4.740073 | 8.76E-05 |
| 3-Methylindole | 0.100323 | 0.986332 | 0.000101 |
| 5-(1,3-dioxo-1,3-dihydro-2H-isoindol-2-yl)-1H-indole-3-carbonitrile | 0.069198 | 14.20572 | 0.000101 |
| 3-(3,4,5-trimethoxyphenyl)propanoic acid | 0.907517 | 0.161421 | 0.000114 |
| Phloretin | 0.087479 | 5.263619 | 0.000115 |
| L-(+)-Citrulline | 0.140976 | 3.666314 | 0.000117 |
| Oleoyl-L-alpha-lysophosphatidic acid | 0.124581 | 0.311719 | 0.000118 |
| Creatinine | 0.79457 | 5.296776 | 0.00012 |
| gamma-Nonanolactone | 2.583595 | 0.379197 | 0.000126 |
| N-Acetylhistidine | 0.228458 | 1.483807 | 0.000145 |
| 3,5-di(2-furylmethylidene)tetrahydro-2H-pyran-4-one | 10.72605 | 2.553205 | 0.000173 |
| 4-Acetamidobutyric Acid | 0.191293 | 0.745975 | 0.000216 |
| MMK | 0.583694 | 0.183153 | 0.000218 |
| Trenbolone acetate | 2.560896 | 0.756126 | 0.000235 |
| Xylitol | 2.014326 | 0.671745 | 0.000256 |
| MNK | 0.042275 | 0.101835 | 0.000257 |
| Cyclohexylsulfamate | 0.13934 | 0.597108 | 0.000273 |
| trans-2-Butene-1,4-dicarboxylic Acid | 0.471351 | 2.789276 | 0.000281 |
| YPH | 0.275832 | 0.078016 | 0.000312 |
| 16-Hydroxyhexadecanoic acid | 354.5146 | 41.09588 | 0.000329 |
| 1,4-Cyclohexanedicarboxylic acid | 5.081024 | 1.344608 | 0.00035 |
| N-gamma-Acetyl-N-2-Formyl-5-Methoxykynurenamine | 0.717573 | 4.603941 | 0.000363 |
| Isobutyryl carnitine | 0.624288 | 1.728532 | 0.000375 |
| Guanine | 0.955482 | 3.132444 | 0.000377 |
| Prostaglandin E1 | 0.773664 | 1.857549 | 0.000389 |
| Nor-9-carboxy-δ9-THC | 10.23864 | 4.382602 | 0.000395 |
| 4-oxododecanedioic acid | 2.042853 | 0.863756 | 0.000409 |
| Guanethidine Monosulfate | 0.12883 | 0.376272 | 0.000415 |
| Lysopc 20:0 | 0.075679 | 0.190297 | 0.000426 |
| O-7460 | 1.324359 | 0.427667 | 0.000439 |
| 3-hydroxy-2-(3-nitro-4-piperidinobenzyl)propanenitrile | 0.193178 | 1.148318 | 0.000483 |
| Methyl N-cyano-N'-[2-(2,2-dichlorocyclopropyl)ethyl]carbamimidothioate | 1.677643 | 0.482016 | 0.000491 |
| N1-isopropyl-2-(1H-2-pyrrolylcarbonyl)-1-hydrazinecarboxamide | 0.804221 | 0.225647 | 0.000506 |
| Mupirocin | 0.264606 | 0.097914 | 0.000515 |
| MAG (18:2) | 2.224593 | 0.413824 | 0.00055 |
| 1-(4-nitrophenyl)piperidine | 0.475522 | 1.071716 | 0.000562 |
| 2,6-Dihydroxybenzoic acid | 0.086026 | 0.233236 | 0.000586 |
| Bz-RS-ISer(3-Ph)-Ome | 0.078703 | 0.837099 | 0.000593 |
| Kinetin 9-riboside | 0.048844 | 0.925193 | 0.000598 |
| Phenylpropiolic acid | 0.444356 | 3.341375 | 0.000601 |
| N-Carbamyl-L-glutamicacid | 1.111039 | 6.221847 | 0.000639 |
| Noradrenaline | 0.498481 | 0.821887 | 0.000649 |
| DL-Tryptophan | 1.845996 | 10.41204 | 0.000657 |
| N-[(4-hydroxy-3-methoxyphenyl)methyl]-8-methylnonanamide | 6.746807 | 2.980458 | 0.000668 |
| Carnosine | 0.762229 | 3.548561 | 0.000691 |
| N alpha-Acetyl-L-Arginine | 0.768918 | 1.801959 | 0.000809 |
| Histamine | 0.561306 | 2.458434 | 0.00081 |
| L-Ornithine | 13.22205 | 5.39662 | 0.000824 |
| L-Dihydroorotic Acid | 0.178425 | 1.326618 | 0.000855 |
| Urocanic acid | 71.4087 | 30.29911 | 0.000888 |
| Ureidosuccinic acid | 1.345522 | 0.419896 | 0.000966 |
| Cyclohexaneacetic acid | 0.895268 | 0.334429 | 0.001016 |
| (±)11-HETE | 3.69589 | 1.4982 | 0.001024 |
| 2-Methoxyestradiol (2-MeOE2) | 16.70278 | 8.293876 | 0.001052 |
| Cryptotanshinone | 11.26312 | 129.9751 | 0.001084 |
| S-Adenosylmethionine | 0.505895 | 2.953445 | 0.00113 |
| PLK | 5.461504 | 2.071768 | 0.001169 |
| 19(R)-Hydroxy-prostaglandin E2 | 19.01791 | 8.465374 | 0.001198 |
| D-δ-Tocopherol | 0.574355 | 0.206918 | 0.001287 |
| 4-(2,3-dihydro-1,4-benzodioxin-6-yl)butanoic acid | 0.35799 | 41.64765 | 0.001302 |
| 5-Hydroxymethyluracil | 0.317838 | 1.237934 | 0.001361 |
| Isorhapontigenin | 0.951589 | 10.21654 | 0.001372 |
| Traumatic acid | 13.2107 | 6.901571 | 0.001383 |
| Cyclic ADP-ribose | 1.258958 | 0.146999 | 0.001383 |
| MPH | 0.394639 | 1.314946 | 0.001398 |
| 6-cyclohex-3-enyl-5-nitropiperidin-2-one | 0.589415 | 3.627688 | 0.001482 |
| TKK | 1.493453 | 0.631078 | 0.001484 |
| (S)-Equol | 0.267977 | 2.369034 | 0.001534 |
| Oxaceprol | 0.528766 | 1.681669 | 0.001551 |
| 5-[(2-hydroxybenzylidene)amino]-2-(2-methoxyethoxy)benzoic acid | 0.076179 | 0.453089 | 0.001649 |
| Deoxycytidine | 1.464912 | 11.40521 | 0.001714 |
| L-Cycloserine | 0.056593 | 0.422686 | 0.001778 |
| Astaxanthin | 1.760014 | 0.516936 | 0.001783 |
| 1-Methyluric acid | 0.209834 | 0.015312 | 0.001811 |
| Homovanillic acid | 0.787769 | 4.104138 | 0.001863 |
| Roquefortine C | 8.675282 | 3.586109 | 0.001894 |
| Piperine | 0.539191 | 6.945625 | 0.001901 |
| 4-Deacetylneosolaniol | 0.819627 | 0.190938 | 0.00191 |
| Ecgonine methyl ester | 0.818807 | 3.784952 | 0.001916 |
| 9,10-Dihome | 1.840301 | 5.937982 | 0.001957 |
| 4-benzylidene-2-phenyltetrahydroisoxazole-3,5-dione | 0.042195 | 0.287833 | 0.002082 |
| (R)-Prunasin | 0.150332 | 1.680607 | 0.002106 |
| 6-Aminonicotinamide | 5.497485 | 2.639672 | 0.002107 |
| 2-Phenylethylamine | 0.134991 | 0.255585 | 0.002145 |
| L-Methionine sulfone | 0.220449 | 0.843559 | 0.002417 |
| Citrulline | 13.03436 | 5.076231 | 0.002473 |
| Delta-Tridecalactone | 0.273818 | 0.58164 | 0.002538 |
| Terephthalic Acid | 0.053664 | 0.08583 | 0.002546 |
| N-(2-morpholinophenyl)-2-furamide | 0.150766 | 0.933705 | 0.002645 |
| Corticosterone | 8.016241 | 2.395061 | 0.002664 |
| Anserine | 0.03705 | 0.191238 | 0.002703 |
| (R)-Equol | 0.402742 | 5.377578 | 0.002763 |
| Guanosine monophosphate (GMP) | 3.328341 | 0.550284 | 0.002808 |
| SMK | 0.300165 | 0.121723 | 0.002821 |
| 1-(4-benzylpiperazino)-2-(pyridin-2-ylamino)propan-1-one | 3.336079 | 0.800545 | 0.002833 |
| Propionyl-L-carnitine | 1.50966 | 0.821183 | 0.002859 |
| ACar 18:1 | 5.865639 | 32.72654 | 0.002922 |
| 4-ethoxy-N-[(4-methoxyphenyl)methylene]aniline | 0.640229 | 1.105168 | 0.00294 |
| 1-(5H-dibenzo[b,f]azepin-5-yl)ethan-1-one | 0.107046 | 0.365552 | 0.002973 |
| 4-(1H-pyrazol-1-yl)-N,N-bis(2-pyridinylmethyl)benzenesulfonamide | 8.569569 | 1.880165 | 0.00305 |
| LysoPE 18:2 | 0.411805 | 1.036304 | 0.003093 |
| JWH 250 N-pentanoic acid metabolite | 1.073734 | 0.371713 | 0.003208 |
| Acipimox | 0.97149 | 7.343364 | 0.003286 |
| MAG (18:4) | 59.60282 | 16.80818 | 0.003339 |
| 16-Heptadecyne-1,2,4-triol | 1.165825 | 2.676741 | 0.003519 |
| D-(-)-Fructose | 28.09252 | 45.33054 | 0.003536 |
| 15(R)-Prostaglandin E2 | 3.266984 | 1.143194 | 0.003631 |
| L-Saccharopine | 1.879223 | 4.038952 | 0.003653 |
| 2-Methylbutyroylcarnitine | 0.600017 | 0.108006 | 0.004063 |
| 5-Hydroxytryptophan | 0.507358 | 0.870093 | 0.004179 |
| 2-Hydroxymyristic acid | 6.67852 | 1.71299 | 0.004441 |
| 2-acetamido-3-(4-methoxyphenyl)propanoic acid | 1.940279 | 4.591468 | 0.004472 |
| Protocatechuic acid | 0.15322 | 0.503324 | 0.004489 |
| Glycerol 1-hexadecanoate | 0.207282 | 0.597007 | 0.004688 |
| (2E)-6-hydroxy-2-methyl-6-(4-methylphenyl)hept-2-enoic acid | 0.409849 | 0.922846 | 0.004723 |
| Royal jelly acid | 6.178413 | 2.771526 | 0.005007 |
| 7-Ketocholesterol | 32.38779 | 11.40895 | 0.005221 |
| DL-α-Methoxyphenylacetic acid | 0.277568 | 0.646473 | 0.005271 |
| Pyrogallol | 1.025334 | 6.023609 | 0.005322 |
| 7-Aminoflunitrazepam-d7 | 0.234739 | 0.064686 | 0.005329 |
| Dl-3-Hydroxy-kynurenine | 0.251543 | 2.137723 | 0.005372 |
| 1a,1b-Dihomo prostaglandin E1 | 19.15487 | 2.613352 | 0.005404 |
| S-(5-Adenosy)-L-Homocysteine | 0.167761 | 0.03414 | 0.005724 |
| 5'-Deoxy-5'-(Methylthio)Adenosine | 0.05556 | 0.406187 | 0.005726 |
| 2,4-Dihydroxybenzoic acid | 0.222845 | 0.888631 | 0.005867 |
| ACar 20:1 | 1.352538 | 6.149875 | 0.006068 |
| 2-[(3S)-1-(3,4-Difluorobenzyl)-3-pyrrolidinyl]-1,3-benzoxazole | 2.237719 | 4.718386 | 0.00611 |
| Hydrocortisone | 1.444746 | 0.616226 | 0.006342 |
| 3-amino-2-phenyl-2H-pyrazolo[4,3-c]pyridine-4,6-diol | 0.909434 | 2.607295 | 0.006361 |
| GNK | 0.184216 | 0.066658 | 0.006496 |
| VPH | 1.162436 | 0.318011 | 0.006516 |
| tetranor-12(R)-HETE | 2.623181 | 1.379384 | 0.006752 |
| Catechol | 0.173677 | 2.196248 | 0.00682 |
| Oxymatrine | 0.517488 | 0.128586 | 0.006834 |
| Lysopc 18:3 | 3.459945 | 1.469529 | 0.006849 |
| Monolaurin | 0.202725 | 0.586162 | 0.006876 |
| Secnidazole | 0.103002 | 0.524659 | 0.006893 |
| 1,7-bis(3,4-dihydroxyphenyl)heptan-3-one | 0.803201 | 3.195926 | 0.006993 |
| RMK | 1.058 | 0.434104 | 0.007017 |
| Hexadecanedioic acid | 23.90197 | 9.559636 | 0.007024 |
| 4-amino-2-(2,4-dimethoxyanilino)-5-pyrimidinecarbonitrile | 9.799991 | 3.164854 | 0.007154 |
| 20-Carboxy-Leukotriene B4 | 8.008552 | 3.552505 | 0.00721 |
| Eicosapentaenoic acid | 5.763161 | 2.677822 | 0.007469 |
| gamma-Glutamylmethionine | 8.117166 | 3.61756 | 0.007569 |
| (R)-3-Hydroxy myristic acid | 6.350949 | 3.336918 | 0.007804 |
| 15-Deoxy-12,14-prostaglandin D2 | 2.913395 | 0.830564 | 0.007913 |
| N-benzoyl-N'-[2-(2-pyridyl)ethyl]thiourea | 0.381187 | 2.166759 | 0.008059 |
| 3-(propan-2-yl)-octahydropyrrolo[1,2-a]pyrazine-1,4-dione | 1.061881 | 7.760397 | 0.008254 |
| 1-Methylhistidine | 0.08526 | 0.151348 | 0.008315 |
| 2-hydroxyiminoindane-1,3-dione | 0.272607 | 0.489438 | 0.008422 |
| (1E)-5-hydroxy-1,7-diphenylhept-1-en-3-one | 0.295716 | 0.110404 | 0.008535 |
| Oxymorphone | 3.829323 | 6.049438 | 0.008623 |
| IKK | 0.71701 | 0.265261 | 0.008999 |
| Azetidine-2-carboxylic acid | 1.394075 | 2.27469 | 0.009044 |
| diethyl 2-[(4-methoxy-2-nitroanilino)methylidene]malonate | 0.7666 | 0.188012 | 0.009126 |
| Thiamine | 3.910365 | 10.06898 | 0.009137 |
| FPK | 1.108848 | 0.282758 | 0.009561 |
| ELK | 6.459996 | 2.083672 | 0.009592 |
| D-Glucosamine 6-phosphate | 27.28897 | 14.6747 | 0.009618 |
| AMK | 0.240203 | 0.049122 | 0.009649 |
| 8-Hydroxyguanosine | 0.126304 | 0.639894 | 0.009801 |
| QNK | 0.353673 | 0.066276 | 0.009861 |
| 23-Nordeoxycholic acid | 37.00128 | 10.18804 | 0.009922 |
| N-Acetylneuraminic acid | 39.31959 | 17.45035 | 0.009954 |
| 5-Hydroxymethyl-2-furancarboxylic acid | 0.350823 | 0.516053 | 0.009981 |
| Adenylosuccinic acid | 0.131371 | 0.01271 | 0.010356 |
| NMK | 0.801129 | 1.752901 | 0.01043 |
| N-[1-(4-methoxy-2-oxo-2H-pyran-6-yl)-2-methylbutyl]acetamide | 27.79887 | 10.37236 | 0.010521 |
| 17beta-Trenbolone | 0.170364 | 0.372752 | 0.010583 |
| 3',5,7-Trihydroxy-4'-methoxyflavanone | 1.984467 | 0.780396 | 0.01063 |
| VLK | 3.463539 | 0.926274 | 0.011 |
| Kahweol | 8.770442 | 2.944407 | 0.011151 |
| GLK | 7.702341 | 2.39984 | 0.011362 |
| Tetranor-PGDM | 1.116297 | 0.617122 | 0.01144 |
| N-Acetyl-L-leucine | 0.722635 | 0.176524 | 0.011494 |
| Pelargonidin | 0.472031 | 5.04187 | 0.011694 |
| (2R)-2-[(2R,5S)-5-[(2S)-2-hydroxybutyl]oxolan-2-yl]propanoic acid | 1.005059 | 0.57991 | 0.011703 |
| ACar 22:6 | 0.313136 | 0.165896 | 0.011867 |
| 2-Thio-acetyl MAGE | 0.281819 | 1.059476 | 0.011895 |
| 1-Methylxanthine | 0.636306 | 0.206484 | 0.011942 |
| 2-Ketoadipic acid | 5.241611 | 10.38284 | 0.012342 |
| Quinolinic acid | 1.015131 | 0.611707 | 0.012373 |
| 17(S)-HpDHA | 38.57855 | 18.37888 | 0.012384 |
| DLK | 8.868539 | 2.796495 | 0.012418 |
| Lysops 22:6 | 7.508058 | 4.150096 | 0.012575 |
| Tyrosol | 0.651156 | 3.335401 | 0.012724 |
| Oxytetracycline | 0.763699 | 0.425733 | 0.012905 |
| 5-Phenylvaleric Acid | 0.109386 | 0.080631 | 0.012939 |
| o-Toluic Acid | 5.274918 | 2.464331 | 0.013011 |
| 3-(5-phenyl-1,3-oxazol-2-yl)-4-(trifluoromethyl)pyridine | 4.671469 | 12.60084 | 0.013671 |
| WLH | 1.215024 | 0.645006 | 0.01407 |
| (5S)-5-hydroxy-1,7-diphenylheptan-3-one | 1.353718 | 3.287574 | 0.014093 |
| Leukotriene B4 Ethanolamide | 3.370908 | 1.379561 | 0.014249 |
| 6-Hydroxymelatonin | 1.961167 | 0.751584 | 0.014309 |
| 4-{3-[(3,4-dihydroxyphenyl)methyl]-2-methylbutyl}benzene-1,2-diol | 0.541828 | 4.535908 | 0.014606 |
| Biopterin | 0.025816 | 0.04565 | 0.01478 |
| 2-(3,4-dihydroxyphenyl)-7-hydroxy-3,4-dihydro-2H-1-benzopyran-4-one | 0.089444 | 0.628491 | 0.014922 |
| QLK | 2.149143 | 0.837123 | 0.015125 |
| Tanespimycin | 0.872784 | 0.332933 | 0.01531 |
| 3-Indoleacrylic acid | 0.293547 | 0.549941 | 0.015528 |
| N-lactoyl-phenylalanine | 0.575086 | 2.797203 | 0.015803 |
| Adenosine 5'-monophosphate | 5.943372 | 0.955489 | 0.015897 |
| Glu-Gln | 2.627034 | 1.563802 | 0.015978 |
| Tetranor-12(S)-HETE | 1.960542 | 0.757359 | 0.016176 |
| MGMG (18:2) | 0.353906 | 0.145698 | 0.01619 |
| N-(2-hydroxyphenyl)acetamide | 0.081165 | 0.377771 | 0.01643 |
| QLH | 1.076196 | 0.275745 | 0.016593 |
| 1-(3,4-dimethoxyphenyl)ethan-1-one oxime | 2.152555 | 3.138648 | 0.016843 |
| Prostaglandin H2 | 6.06401 | 3.653797 | 0.016864 |
| Cytidine | 1.747692 | 0.642787 | 0.017312 |
| 3-Methoxyphenylacetic acid | 0.403968 | 2.471706 | 0.017446 |
| PNK | 3.763604 | 1.995919 | 0.017508 |
| ACar 15:0 | 0.385594 | 1.790111 | 0.017568 |
| LPH | 2.121422 | 1.179517 | 0.017872 |
| beta-Nicotinamide mononucleotide | 0.344732 | 0.593775 | 0.017899 |
| 12-Oxo phytodienoic acid | 0.819353 | 0.416078 | 0.01809 |
| Estradiol Benzoate | 0.203195 | 0.089815 | 0.01828 |
| 3'-Adenosine monophosphate (3'-AMP) | 1.771007 | 0.196589 | 0.018387 |
| Palmitoylcarnitine | 4.036037 | 39.42131 | 0.018401 |
| Metanephrine | 0.223057 | 0.664026 | 0.01845 |
| Oxohongdenafil | 0.88941 | 0.322083 | 0.018516 |
| 5-oxo-3-phenyl-5-(2-quinolinylamino)pentanoic acid | 1.283232 | 0.668353 | 0.018953 |
| INK | 4.878605 | 2.047815 | 0.018957 |
| Genistein | 0.3656 | 4.78493 | 0.018963 |
| 4-Hydroxyretinoic Acid | 2.409891 | 0.765101 | 0.019082 |
| Prostaglandin K2 | 5.592141 | 0.914974 | 0.019235 |
| ILK | 7.832322 | 2.25894 | 0.019434 |
| Chlorindione | 0.086196 | 0.234657 | 0.019543 |
| Naringenin | 0.10866 | 1.511967 | 0.019655 |
| 2,3-Dinor-11β-prostaglandin F2α | 6.589105 | 2.791525 | 0.019889 |
| IMK | 2.242382 | 0.645725 | 0.020198 |
| Ala-Gln | 1.023162 | 0.168753 | 0.020335 |
| Cortisol | 2.062109 | 1.599551 | 0.02035 |
| 4-(acetylamino)phenyl 3-chlorobenzoate | 0.337184 | 0.756291 | 0.020453 |
| Dehydrocholic acid | 8.905954 | 4.209209 | 0.021392 |
| L-Ascorbic acid 2-sulfate | 5.728148 | 0.714569 | 0.021495 |
| beta-Estradiol 17-Acetate | 2.066836 | 0.681201 | 0.021528 |
| TLK | 5.636613 | 2.102827 | 0.02176 |
| DKK | 0.118081 | 0.056498 | 0.021808 |
| LNH | 0.49365 | 0.177907 | 0.021869 |
| ANK | 0.314642 | 0.514744 | 0.02213 |
| EMK | 1.365547 | 0.81515 | 0.022551 |
| Maltotetraose | 0.436228 | 0.829566 | 0.022959 |
| β-Cortolone | 0.511915 | 0.17724 | 0.023022 |
| 6β-Hydroxycortisol | 4.166554 | 2.016804 | 0.023367 |
| HNK | 0.565859 | 0.330973 | 0.0237 |
| 2-Arachidonoyl glycerol | 0.349149 | 18.33263 | 0.024421 |
| GPK | 1.270127 | 0.856925 | 0.024905 |
| 1-Methyladenosine | 2.743447 | 3.89464 | 0.025022 |
| Sorbitan monopalmitate | 2.130682 | 0.912298 | 0.025665 |
| N,N-dimethyl-5-nitro-6-[3-(trifluoromethyl)phenoxy]pyrimidin-4-amine | 0.038747 | 0.10304 | 0.025691 |
| Ursolic acid | 1.452324 | 10.65784 | 0.025954 |
| Amino adipic acid | 0.910904 | 0.510124 | 0.026345 |
| Nootkatone | 0.733386 | 2.126926 | 0.026353 |
| 4-Methoxycinnamic Acid | 0.243546 | 0.970701 | 0.027743 |
| Nicotinate ribonucleoside | 0.548979 | 1.929464 | 0.029251 |
| Uridine monophosphate (UMP) | 4.378277 | 1.452207 | 0.029616 |
| SLK | 4.795402 | 1.829367 | 0.030164 |
| Adenine | 0.792912 | 1.460212 | 0.030249 |
| 4-phenoxyphenyl 4-hydroxypiperidine-1-carboxylate | 0.093914 | 0.062341 | 0.030268 |
| Glycitein | 0.677491 | 5.514004 | 0.030974 |
| EPK | 0.232345 | 0.10862 | 0.031187 |
| 7-(2-hydroxypropan-2-yl)-1,4a-dimethyl-decahydronaphthalen-1-ol | 7.795277 | 4.703085 | 0.031223 |
| ALK | 7.373226 | 2.270008 | 0.031452 |
| 8-iso Prostaglandin A2 | 2.0006 | 0.866813 | 0.032763 |
| (+)-alpha-Lipoic acid | 5.634871 | 3.882776 | 0.032787 |
| D-Sedoheptulose 7-phosphate | 1.010099 | 0.565325 | 0.033069 |
| NG,NG-Dimethyl-L-arginine | 1.593937 | 0.714843 | 0.033075 |
| dopaquinone | 2.803951 | 0.328271 | 0.033297 |
| 13(S)-HOTrE | 4.014039 | 15.49398 | 0.03348 |
| 2-(14,15-Epoxyeicosatrienoyl) glycerol | 0.208075 | 3.320136 | 0.03372 |
| 10-Nitrolinoleate | 1.140495 | 0.737947 | 0.033946 |
| Myricetin | 1.308196 | 0.503309 | 0.034008 |
| Lagochilin | 0.325999 | 1.108557 | 0.03412 |
| GKK | 0.04707 | 0.027565 | 0.034367 |
| Artemisinin | 1.037289 | 0.5343 | 0.034543 |
| 4-(Diethylamino)salicylaldehyde | 1.087485 | 0.638293 | 0.03495 |
| 5-Hydroxyindole-3-acetic acid | 4.271377 | 15.09231 | 0.035552 |
| tetranor-PGFM | 1.025693 | 2.532423 | 0.03601 |
| Tyramine | 0.61496 | 0.305974 | 0.036091 |
| ACar 17:0 | 0.735374 | 2.277517 | 0.036369 |
| Flunitrazepam-d3 | 0.853384 | 1.491052 | 0.037081 |
| Tretinoin | 3.637584 | 1.022784 | 0.037516 |
| 3-[(4-chlorophenyl)thio]-1-(3-pyridylmethyl)pyrrolidine-2,5-dione | 2.398999 | 1.213624 | 0.038206 |
| Corchorifatty acid F | 1.809325 | 5.817407 | 0.038269 |
| N-acetyl-D-glucosamine | 79.75815 | 46.57791 | 0.03833 |
| FAHFA (3:0/24:1) | 18.01452 | 5.741458 | 0.039254 |
| Avocadyne 1-acetate | 1.77002 | 13.319 | 0.039912 |
| 19(R)-hydroxy Prostaglandin A2 | 5.425832 | 1.86442 | 0.040293 |
| α-Hydroxyhippuric acid | 0.301081 | 0.595923 | 0.04041 |
| 8-iso-15-keto Prostaglandin F2α | 4.037537 | 1.835034 | 0.040506 |
| Orsellinic acid ethyl ester | 1.079468 | 3.396062 | 0.04126 |
| Esculin | 0.650452 | 0.307213 | 0.041725 |
| 3-pentadecyl-4,5,6,7-tetrahydrobenzo[d]isoxazol-4-one oxime | 0.878495 | 2.890419 | 0.042245 |
| KPH | 0.243343 | 0.730025 | 0.042367 |
| Noroxycodone-d3 | 0.371281 | 1.531286 | 0.042462 |
| Glycolithocholic acid | 0.256129 | 0.885556 | 0.04275 |
| 7-Methylxanthine | 0.315899 | 0.134945 | 0.042845 |
| LPE 20:5 | 0.579061 | 5.503074 | 0.042974 |
| N-Acetyl-L-tyrosine | 0.688536 | 1.075928 | 0.04317 |
| Glycyl-L-leucine | 3.856507 | 1.619535 | 0.043211 |
| Norfloxacin | 4.444448 | 6.882085 | 0.043396 |
| 5-fluoro AB-PINACA N-(4-hydroxypentyl) metabolite | 0.075201 | 1.218058 | 0.045077 |
| Dehydroepiandrosterone (DHEA) | 35.72118 | 24.15529 | 0.045128 |
| S-(Methyl)Glutathione | 0.174422 | 0.334187 | 0.045906 |
| 5,6-dihydroxyindole | 0.317576 | 0.707063 | 0.046522 |
| 5-S-cysteinyldopaquinone | 0.678863 | 1.743806 | 0.04683 |
| Ethyl-β-D-glucuronide | 6.319337 | 2.879561 | 0.047744 |
| Ip7G | 2.30608 | 0.453437 | 0.048089 |
| N-Acetylmannosamine | 85.18559 | 53.15769 | 0.048169 |
| Ferulic acid | 0.422249 | 2.129462 | 0.048731 |
| L-(-)-alpha-Amino-epsilon-Caprolactam | 2.580989 | 1.226835 | 0.04964 |
